# The relationship between sarcopenia and mortality in Chinese community-dwelling adults: a 7-year cohort study with propensity score matching and Mendelian randomization

**DOI:** 10.3389/fendo.2023.1215512

**Published:** 2023-10-04

**Authors:** Lijiao Xiong, Tingfeng Liao, Tianting Guo, Zhaohao Zeng, Shuojia Wang, Guangyan Yang, Xiaohao Wang, Xinyu Wang, Jing Zhu, Pengfei Zhao, Yanchun Li, Lixing Li, Lin Kang, Shu Yang, Zhen Liang

**Affiliations:** ^1^ Department of Geriatrics, The Second Clinical Medical College, Jinan University (Shenzhen People’s Hospital, The First Affiliated Hospital of Southern University of Science and Technology), Shenzhen, China; ^2^ Guangdong Provincial Clinical Research Center for Geriatrics, Shenzhen Clinical Research Center for Geriatrics, Shenzhen People’s Hospital (The Second Clinical Medical College, Jinan University, The First Affiliated Hospital, Southern University of Science and Technology), Shenzhen, China; ^3^ Department of Orthopedics, Ganzhou Hospital of Guangdong Provincial People’s Hospital, Ganzhou Municipal Hospital, Ganzhou, China; ^4^ Department of Neurology, Shenzhen People’s Hospital (The Second Clinical Medical College, Jinan University, The First Affiliated Hospital, Southern University of Science and Technology), Shenzhen, China; ^5^ Post-doctoral Scientific Research Station of Basic Medicine, Jinan University, Guangzhou, China

**Keywords:** the CHARLS, sarcopenia, mortality risk, the propensity score matching, Mendelian randomization

## Abstract

**Background:**

Sarcopenia has been linked to adverse health outcomes, including an increased risk of mortality. This study aimed to assess the 7-year mortality risk of sarcopenia in a community-based population in China and explore the causal relationship between components of sarcopenia and any death.

**Methods:**

Data were sourced from the China Health and Retirement Longitudinal Study (CHARLS) conducted between 2011 and 2018. Sarcopenia was diagnosed using the Asian Working Group for Sarcopenia (AWGS) 2019 criteria. Logistic regression, Kaplan–Meier (KM) survival analysis, and propensity score matching with inverse probability of treatment weighting were used. Mendelian randomization (MR) analyses, conducted using European population data, were utilized to assess causality between sarcopenia and any death.

**Results:**

The study included 9,006 participants: 3,892 had no sarcopenia, 3,570 had possible sarcopenia, 1,125 had sarcopenia, and 419 had severe sarcopenia. Over 7 years of follow-up, there were 871 deaths, including 196 with sarcopenia and 133 with severe sarcopenia. The KM curves showed that sarcopenia had a higher risk of mortality. Compared to those of no sarcopenia, the odds ratios (ORs) of sarcopenia for 7-year mortality were 1.41 (95% CI, 1.06–1.87) after adjusting for confounding variables (*p* < 0.05). The ORs of severe sarcopenia were 2.11 (95% CI, 1.51–2.95). Propensity score matching analysis and inverse probability of treatment weighting analysis confirmed these findings. The adjusted ORs of sarcopenia and 7-year mortality were 2.94 (95% CI, 1.6–5.39) in the 45–60 age group, 1.72 (95% CI, 1.11–2.68) in the 60–80 age group, and 5.03 (95% CI, 0.48–52.65) in the ≥80 age group. The ORs of severe sarcopenia and 7-year mortality were 6.92 (95% CI, 1.95–24.5) in the 45–60 age group, 2.59 (95% CI, 1.61–4.17) in the 60–80 age group, and 12.52 (95% CI, 1.18–133.18) in the ≥80 age group. The MR analyses, leveraging the inverse variance weighted (IVW) method, unveiled substantial causal links between low hand grip strength in individuals aged 60 and older, the usual walking pace, and mortality risk.

**Conclusion:**

This study underscores the significant impact of sarcopenia and its components on mortality risk within the Chinese population. Particularly, low hand grip strength and usual walking pace emerged as noteworthy contributors to mortality risk.

## Introduction

Sarcopenia, characterized by loss of muscle mass and function, is a common condition in older adults that has been associated with increased disability, falls, hospitalization, and mortality (1–5). It has been reported to be prevalent in various groups, with estimates ranging from 9.9% to 40.4% among community-dwelling adults and 2% to 34% in outpatient settings, and affects as many as 56% of hospitalized patients (6–9). As the global population ages, the prevalence of sarcopenia is expected to increase significantly (10). Despite its growing recognition as a significant public health issue, there are limited data on the association between sarcopenia and mortality risk in Chinese community-dwelling adults. Moreover, the relationship between sarcopenia and mortality can be confounded by chronic diseases and other factors that commonly occur with aging. Propensity score matching has been used in previous research to account for these confounding factors, but typically for a single disease (11–14).

Sarcopenia is linked to a doubling of mortality risk in both community-dwelling adults and nursing home residents and a tripling of risk in cancer patients (11–14). As the world’s population continues to age, addressing the health implications of sarcopenia has become a critical priority. However, despite the increasing recognition of sarcopenia’s importance, there are still gaps in our understanding of its causal relationship with mortality.

This study aims to contribute to this understanding by investigating the causal links between different components of sarcopenia—specifically, appendicular lean mass, low hand grip strength, and usual walking pace—and the risk of mortality in a comprehensive manner. Our research combines data from a longitudinal study conducted among Chinese adults aged 45 years and older with a Mendelian randomization study utilizing European population data. By applying Mendelian randomization methods, we can better elucidate the causal relationships between these sarcopenia components and mortality risk.

## Methods

### Longitudinal study

#### Population

**Figure 1:**
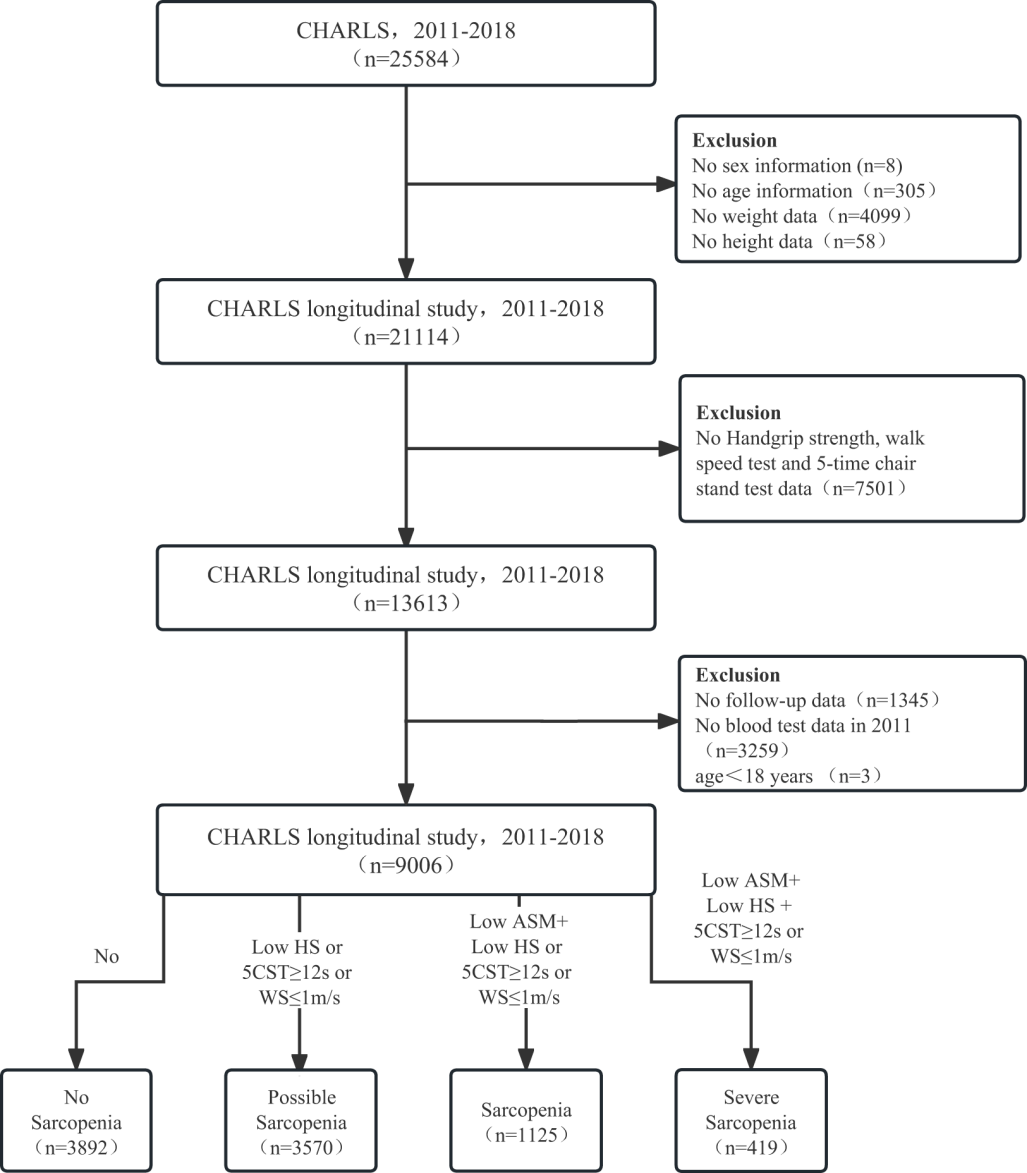
The flow diagram for the population in the CHARLS. CHARLS, The China Health and Retirement Longitudinal Study; HS, handgrip strength; CST, 5-time chair stand tests; WS, walk speed; ASM, appendicular skeletal muscle mass.

The China Health and Retirement Longitudinal Study (CHARLS), established in 2011, is a national longitudinal study of community-dwelling adults in China, with its detailed validity and methodology previously documented (15, 16). The CHARLS protocol was approved by the Peking University Ethical Review Committee (IRB00001052-11015) following the Declaration of Helsinki. Informed consent was obtained from all participants. Data from Harmonized CHARLS 2011–2018 were included. Missing data on sex (n = 8), age (n = 305), weight (n = 4,099), height (n = 58), no-grip strength, walking speed, sitting test (n = 7,501), no follow-up (n = 1,345), blood sample data (n = 3,259), and age < 18 years (n = 3) were excluded. A total of 9,006 participants (≥18 years) were enrolled in the study (**Figure 1**).

#### Evaluation of sarcopenia status

Asian Working Group for Sarcopenia (AWGS) 2019 algorithm was used to evaluate sarcopenia status in the CHARLS, including assessment of muscle mass, muscle strength, and physical performance (1). The muscle mass values, the appendicular skeletal muscle mass (ASM), were imputed using an anthropometric equation (ASM = 0.193 * body weight + 0.107 * height − 4.157 * sex − 0.037 * age − 2.631) mainly validated in Asia populations as described in previous studies (17, 18). The handgrip strength of the dominant hand was recorded by a Yuejian TM WL-1000 dynamometer. The participants carried out the gait speed and five-time chair stand tests, and the methods employed in the CHARLS have been described (15, 17).

#### Mortality data

From April 2011 to March 2019, all deaths were recorded, and the survival status of the participants was determined during the baseline investigation in 2011–2012, prior to follow-up. Death data were collected from life history surveys conducted in 2013, 2014, 2015, and 2018, with the follow-up period spanning approximately 8 years. The survival status of participants was ascertained through field investigations conducted by interviewers during four separate follow-up periods. Interviewers visited the residences of participants and, in the event of the participant’s death, collected relevant information by interviewing household members who lived with the deceased (19).

#### Covariates

Sociodemographic and medical covariable data were extracted from the CHARLS 2011. These variables included age, gender (male or female), marital status (single, married, divorced, or widowed or others), education (elementary school or less, or secondary school or above), dwelling locations (urban or rural), drinking, smoking, and multimorbidity (20). The multimorbidity covariate consisted of self-reported data on 12 medically diagnosed conditions, including hypertension, diabetes, cancer, chronic lung diseases, liver disease, heart disease, stroke, kidney disease, digestive diseases, memory-related diseases, arthritis or rheumatoid arthritis, and asthma (20, 21).

#### Statistical analysis

For continuous variables, confidence intervals (CIs) of 95% were supplied, whereas percentage frequencies were provided for categorical variables. To compare continuous and categorical data, *t*-tests and χ^2^ were utilized. With the use of logistic regression models, the risk of mortality is determined. The Kaplan–Meier curves are depicted visually. To reduce potential selection bias, propensity score matching (PSM) was utilized to balance covariates between participants with and without sarcopenia or severe sarcopenia. After individual propensity scores were computed using a logistic regression model, the nearest-neighbor matching technique with a caliper width of 0.2 standard deviations of the propensity score was used to pair patients from the lowest hand grip strength (HGS) group with those from other groups. Then, a regression analysis was conducted using the inverse probability of treatment weighting (IPTW) (22). Statistical analyses were carried out using the R software package (http://www.R-project.org, The R Foundation) and the Free Statistics software version 1.7. Statistical significance was determined by a two-sided *p*-value <0.05.

### Mendelian randomization study

#### Data source

The Mendelian randomization study was conducted using European populations to examine the causal relationship between sarcopenia and any death. The exposures analyzed were appendicular lean mass, low hand grip strength in those aged 60 years and older, and usual walking pace. Appendicular lean mass data were obtained in 2020 from 205,513 samples genotyped for 18,164,071 single-nucleotide polymorphisms (SNPs) (https://gwas.mrcieu.ac.uk/datasets/ebi-a-GCST90000026/). Low hand grip strength was analyzed in 2021 using 256,523 samples genotyped for 9,336,415 SNPs (https://gwas.mrcieu.ac.uk/datasets/ebi-a-GCST90007526/). The usual walking pace was examined in 2018 with 459,915 samples genotyped for 9,851,867 SNPs (https://gwas.mrcieu.ac.uk/datasets/ukb-b-4711/). The outcome was any cause mortality, analyzed in 2021 with 218,792 samples genotyped for 16,380,466 SNPs (https://gwas.mrcieu.ac.uk/datasets/finn-b-DEATH/). Sample sizes ranged from 205,513 to 459,915; SNPs analyzed spanned 9,336,415 to 18,164,071; years of data collection were from 2018 to 2021 (**Supplementary Table 1**).

#### Selection of SNPs and statistical analysis

The Mendelian randomization (MR) analysis was conducted employing the inverse variance weighted (IVW) method. The SNP selection process involved several methodologies: a significance threshold of *p* < 5 × 10^−8^ was applied to identify SNPs, achieving genome-wide significance. A threshold of r^2^ < 0.001 (with a clumping distance of 10,000 kb) was set to exclude SNPs that were in a state of linkage disequilibrium. In addressing potential pleiotropy, the PhenoScanner database was utilized for SNP identification and subsequent exclusion. Weak instrumental variables, as indicated by an F-statistic < 10, were systematically excluded from the analysis. The MR–pleiotropy residual sum and outlier (MR-PRESSO) method was employed before each MR analysis to eliminate potential outliers. Additionally, palindromic SNPs were eliminated through data harmonization between the any death dataset and the exposure dataset. Following this meticulous screening process, the remaining SNPs were retained for subsequent analyses. To validate the findings, alternative methods including MR-Egger, weighted median, and weighted mode were applied alongside IVW. Heterogeneity was assessed using Cochran’s Q test, while pleiotropy was evaluated through MR-Egger intercept testing and leave-one-out analysis. All statistical analyses were conducted using R software (version 4.3.0) for both MR analyses and sensitivity assessments.

## Results

### Longitudinal study

#### Demographics

The study included 9,006 participants, of which 45.9% were male and 54.1% were female. Among them, 3,892 had no sarcopenia, 3,570 had possible sarcopenia, 1,125 had sarcopenia, and 419 had severe sarcopenia. Over 7 years of follow-up, there were 871 deaths, including 147 without sarcopenia, 395 with possible sarcopenia, 196 with sarcopenia, and 133 with severe sarcopenia (**Table 1**).

**Table 1:** The baseline characteristics of the study population in the CHARLS.

Characteristic	Total (n = 9,006)	No sarcopenia (n = 3,892)	Possible sarcopenia (n = 3,570)	Sarcopenia (n = 1,125)	Severe arcopenia (n = 419)	*p*-Value
Age, years	58.8 ± 9.6	52.3 ± 6.2	61.8 ± 8.3	67.1 ± 8.3	71.4 ± 8.1	<0.001
Gender, n (%)						0.002
Male	4,138 (45.9)	1,798 (46.2)	1,693 (47.4)	461 (41)	186 (44.4)	
Female	4,868 (54.1)	2,094 (53.8)	1,877 (52.6)	664 (59)	233 (55.6)	
BMI (kg/m^2^)	23.5 ± 3.9	24.0 ± 3.9	24.8 ± 3.4	19.3 ± 1.8	19.3 ± 1.9	<0.001
Weight (kg)	58.7 ± 11.7	61.1 ± 11.6	61.9 ± 9.6	45.8 ± 5.5	44.4 ± 5.5	<0.001
Height (m)	1.6 ± 0.1	1.6 ± 0.1	1.6 ± 0.1	1.5 ± 0.1	1.5 ± 0.1	<0.001
ASM (kg)	17.0 ± 4.2	17.9 ± 4.2	17.6 ± 3.7	13.6 ± 3.6	13.0 ± 3.5	<0.001
ASM/Ht^2^ (kg/m^2^)	6.7 ± 1.1	7.0 ± 1.1	7.0 ± 1.0	5.6 ± 0.9	5.6 ± 1.0	<0.001
Walk speed (m/s)	0.6 ± 0.2	1.1 ± 0.1	0.6 ± 0.2	0.6 ± 0.2	0.5 ± 0.2	<0.001
Handgrip strength (kg)	31.7 ± 10.4	35.6 ± 9.7	30.1 ± 9.9	28.1 ± 7.7	17.5 ± 5.9	<0.001
5-time chair stand test (s)	10.8 ± 4.3	8.5 ± 1.9	12.7 ± 4.8	12.0 ± 4.0	14.1 ± 6.3	<0.001
Waist (cm)	84.3 ± 12.6	84.5 ± 11.9	87.7 ± 12.7	75.7 ± 9.2	75.6 ± 10.5	<0.001
Education, n (%)						<0.001
Elementary school or below	6,412 (71.2)	2,258 (58)	2,776 (77.8)	982 (87.3)	396 (94.5)	
Secondary school	2,506 (27.8)	1,590 (40.9)	756 (21.2)	138 (12.3)	22 (5.3)	
College or above	88 (1.0)	44 (1.1)	38 (1.1)	5 (0.4)	1 (0.2)	
Marriage, n (%)						<0.001
Single	57 (0.6)	18 (0.5)	22 (0.6)	7 (0.6)	10 (2.4)	
Married	7,907 (87.8)	3,664 (94.1)	3,082 (86.3)	875 (77.8)	286 (68.3)	
Divorced or widowed or others	1,042 (11.6)	210 (5.4)	466 (13.1)	243 (21.6)	123 (29.4)	
Area, n (%)						<0.001
Urban area	2,972 (33.0)	1,406 (36.1)	1,209 (33.9)	251 (22.3)	106 (25.3)	
Rural area	6,034 (67.0)	2,486 (63.9)	2,361 (66.1)	874 (77.7)	313 (74.7)	
Drinking, n (%)						<0.001
No	6,543 (72.7)	2,665 (68.5)	2,665 (74.6)	885 (78.7)	328 (78.3)	
Yes	2,463 (27.3)	1,227 (31.5)	905 (25.4)	240 (21.3)	91 (21.7)	
Smoking, n (%)						0.261
No	6,307 (70.0)	2,694 (69.2)	2,542 (71.2)	783 (69.6)	288 (68.7)	
Yes	2,699 (30.0)	1,198 (30.8)	1,028 (28.8)	342 (30.4)	131 (31.3)	
Diabetes, n (%)						<0.001
No	7,495 (83.2)	3,326 (85.5)	2,834 (79.4)	989 (87.9)	346 (82.6)	
Yes	1,511 (16.8)	566 (14.5)	736 (20.6)	136 (12.1)	73 (17.4)	
Hypertension, n (%)						<0.001
No	6,640 (73.7)	3,134 (80.5)	2,276 (63.8)	898 (79.8)	332 (79.2)	
Yes	2,366 (26.3)	758 (19.5)	1,294 (36.2)	227 (20.2)	87 (20.8)	
Cancer, n (%)						0.878
No	8,922 (99.1)	3,854 (99)	3,535 (99)	1,117 (99.3)	416 (99.3)	
Yes	84 (0.9)	38 (1)	35 (1)	8 (0.7)	3 (0.7)	
Heart disease, n (%)						<0.001
No	7,951 (88.3)	3,557 (91.4)	3,011 (84.3)	1,011 (89.9)	372 (88.8)	
Yes	1,055 (11.7)	335 (8.6)	559 (15.7)	114 (10.1)	47 (11.2)	
Stroke, n (%)						<0.001
No	8,772 (97.4)	3,827 (98.3)	3,445 (96.5)	1,099 (97.7)	401 (95.7)	
Yes	234 (2.6)	65 (1.7)	125 (3.5)	26 (2.3)	18 (4.3)	
Lung disease, n (%)						<0.001
No	8,131 (90.3)	3,643 (93.6)	3,192 (89.4)	945 (84)	351 (83.8)	
Yes	875 (9.7)	249 (6.4)	378 (10.6)	180 (16)	68 (16.2)	
Arthre disease, n (%)						<0.001
No	5,847 (64.9)	2,700 (69.4)	2,174 (60.9)	722 (64.2)	251 (59.9)	
Yes	3,159 (35.1)	1,192 (30.6)	1,396 (39.1)	403 (35.8)	168 (40.1)	
Liver disease, n (%)						0.999
No	8,818 (97.9)	3,810 (97.9)	3,496 (97.9)	1,102 (98)	410 (97.9)	
Yes	188 (2.1)	82 (2.1)	74 (2.1)	23 (2)	9 (2.1)	
Kidney disease, n (%)						0.017
No	8,495 (94.3)	3,699 (95)	3,334 (93.4)	1,067 (94.8)	395 (94.3)	
Yes	511 (5.7)	193 (5)	236 (6.6)	58 (5.2)	24 (5.7)	
Digestive disease, n (%)						0.042
No	6,955 (77.2)	3,037 (78)	2,764 (77.4)	849 (75.5)	305 (72.8)	
Yes	2,051 (22.8)	855 (22)	806 (22.6)	276 (24.5)	114 (27.2)	
Asthma, n (%)						<0.001
No	8,591 (95.4)	3,781 (97.1)	3,378 (94.6)	1,043 (92.7)	389 (92.8)	
Yes	415 (4.6)	111 (2.9)	192 (5.4)	82 (7.3)	30 (7.2)	
Memory-related disease, n (%)						<0.001
No	8,874 (98.5)	3,873 (99.5)	3,490 (97.8)	1,106 (98.3)	405 (96.7)	
Yes	132 (1.5)	19 (0.5)	80 (2.2)	19 (1.7)	14 (3.3)	
7-year mortality, n (%)						<0.001
No	8,135 (90.3)	3,745 (96.2)	3,175 (88.9)	929 (82.6)	286 (68.3)	
Yes	871 (9.7)	147 (3.8)	395 (11.1)	196 (17.4)	133 (31.7)	

CHARLS, China Health and Retirement Longitudinal Study; BMI, body mass index; ASM, appendicular skeletal muscle mass.

#### Univariate and multivariate logistic regression analyses

The univariate and multivariate logistic regression analyses showed that the risk factors for 7-year mortality were sarcopenia (odds ratios (OR): 1.41, 95% CI, 1.06–1.87), severe sarcopenia (OR = 2.11, 95% CI, 1.51–2.95), age (OR = 1.09, 95% CI, 1.08–1.1), diabetes (OR = 1.51, 95% CI, 1.26 to 1.82), hypertension (OR = 1.31, 95% CI, 1.11 to 1.56), cancer (OR = 4.46, 95% CI, 2.45 to 8.09), stroke (OR = 1.64, 95% CI, 1.13 to 2.38), lung disease (OR = 1.82, 95% CI, 1.45 to 2.28), and memory-related diseases (OR = 2.11, 95% CI, 1.37–3.25) (*p* < 0.05). The protective factors for 7-year mortality were female (OR = 0.52, 95% CI, 0.43–0.63), married (OR = 0.33, 95% CI, 0.17–0.61), and divorced (OR = 0.46, 95% CI, 0.24–0.9) (*p* < 0.05) (**Table 2**).

**Table 2:** Univariate and multivariate logistic analyses of risk factors for 7-year mortality in the CHARLS.

Variable	Univariate logistic regression		Multivariate logistic regression	
	OR (95% CI)	*p*-Value	OR (95% CI)	*p*-Value
Possible sarcopenia	3.17 (2.61–3.85)	<0.001	1.11 (0.89–1.4)	0.356
Sarcopenia	5.37 (4.29–6.74)	<0.001	1.41 (1.06–1.87)	0.017
Severe sarcopenia	11.85 (9.1–15.42)	<0.001	2.11 (1.51–2.95)	<0.001
Age, years	1.11 (1.1–1.12)	<0.001	1.09 (1.08–1.1)	<0.001
Female	0.5 (0.44–0.58)	<0.001	0.52 (0.43–0.63)	<0.001
Education Secondary school	0.46 (0.38–0.55)	<0.001	0.83 (0.67–1.03)	0.097
Education College or above	0.47 (0.19–1.16)	0.103	0.53 (0.2–1.37)	0.19
Married	0.21 (0.12–0.37)	<0.001	0.33 (0.17–0.61)	0.001
Divorced or widowed or others	0.61 (0.34–1.09)	0.097	0.46 (0.24–0.9)	0.022
Rural area	1.13 (0.97–1.31)	0.122	1 (0.84–1.18)	0.976
Drinking	1.02 (0.88–1.2)	0.761	0.98 (0.82–1.18)	0.852
Smoking	1.41 (1.22–1.63)	<0.001	1.12 (0.93–1.35)	0.237
Diabetes	1.67 (1.41–1.97)	<0.001	1.51 (1.26–1.82)	<0.001
Hypertension	1.64 (1.42–1.9)	<0.001	1.31 (1.11–1.56)	0.002
Cancer	2.77 (1.65–4.64)	<0.001	4.46 (2.45–8.09)	<0.001
Heart disease	1.5 (1.23–1.82)	<0.001	1.24 (0.99–1.54)	0.062
Stroke	2.56 (1.85–3.54)	<0.001	1.64 (1.13–2.38)	0.009
Lung disease	2.47 (2.05–2.97)	<0.001	1.82 (1.45–2.28)	<0.001
Arthre disease	0.99 (0.85–1.14)	0.851	0.89 (0.76–1.05)	0.173
Kidney disease	1.34 (1.02–1.76)	0.037	1.29 (0.95–1.75)	0.107
Digestive disease	0.86 (0.72–1.02)	0.084	0.92 (0.76–1.12)	0.407
Asthma	1.9 (1.45–2.49)	<0.001	0.87 (0.63–1.21)	0.411
Memory-related disease	4.05 (2.77–5.93)	<0.001	2.11 (1.37–3.25)	0.001

CHARLS, China Health and Retirement Longitudinal Study; OR, odds ratio.

#### The relationship between sarcopenia and all-cause mortality

**Figure 2:**
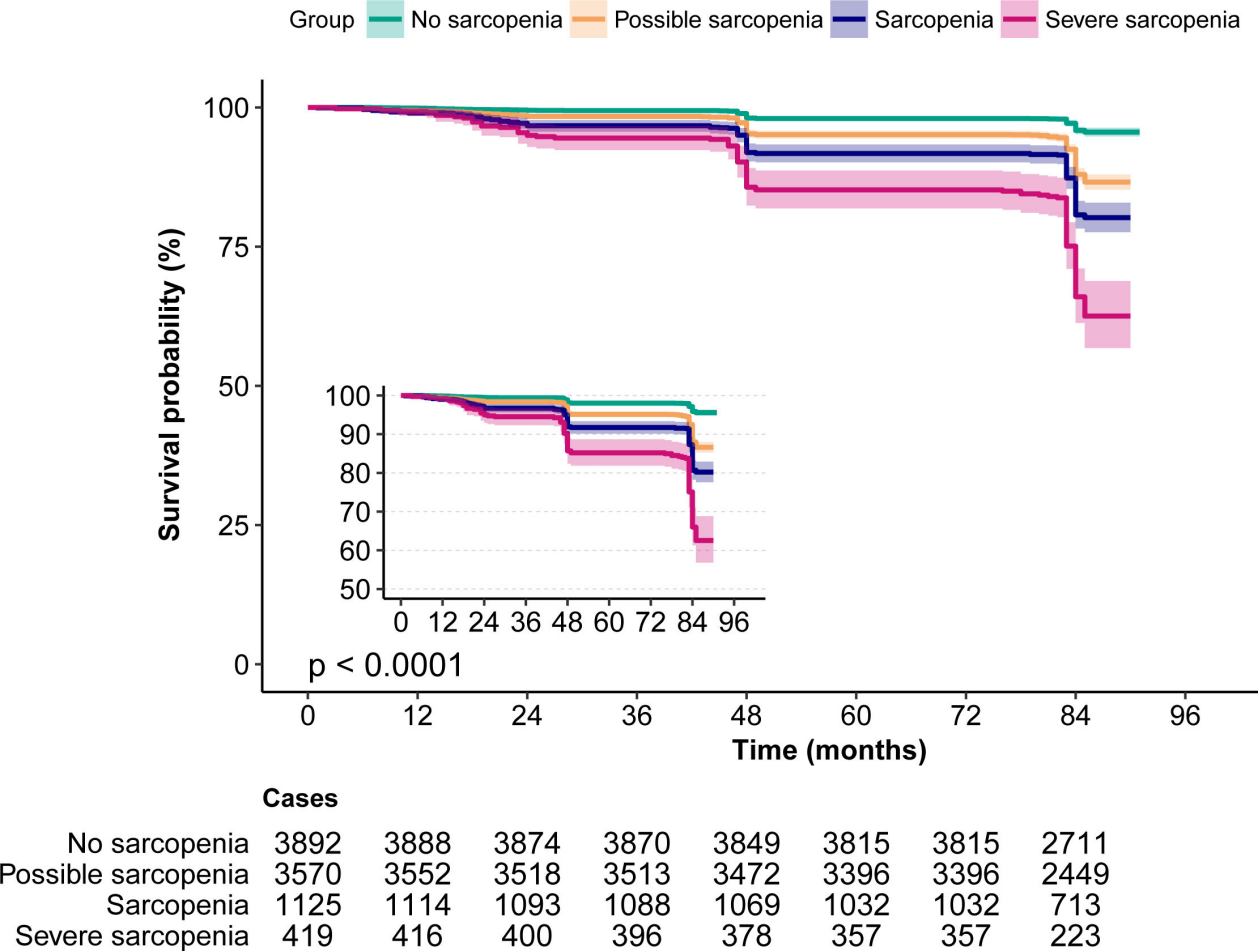
The Kaplan–Meier survival curves for sarcopenia associated with all-cause mortality risk. Note the small Kaplan–Meier graph on the left and its enlarged view.

According to the Kaplan–Meier survival curves, sarcopenia and severe sarcopenia had a higher risk of 7-year mortality (**Figure 2**). The results from logistic regression analyses are presented in **Table 2**. Compared to no sarcopenia, the unadjusted OR of sarcopenia for 7-year mortality (model 1) was 5.37 (95% CI, 4.29–6.74), whereas for severe sarcopenia, it was 11.85 (95% CI, 9.1–15.42). After adjusting for age and sex (model 2), the OR of sarcopenia and severe sarcopenia for 7-year mortality was 1.41 (95% CI, 1.07–1.85) and 2.2 (95% CI, 1.59–3.05). Further adjusting for age, sex, education level, marriage status, urban area, drinking, and smoking (model 3), the OR of sarcopenia and severe sarcopenia for 7-year mortality was 1.39 (95% CI, 1.06–1.83) and 2.1 (95% CI, 1.51–2.92). Finally, after adjusting for age, body mass index (BMI), education level, marriage status, urban area, drinking, smoking, diabetes, hypertension, cancer, heart disease, stroke, lung disease, Arthre disease, liver disease, kidney disease, digestive disease, asthma, and memory-related disease (model 4), the OR of sarcopenia and severe sarcopenia for 7-year mortality was 1.41 (95% CI, 1.06–1.87) and 2.11 (95% CI, 1.51–2.95). All of the unadjusted and adjusted ORs of sarcopenia and severe sarcopenia were statistically significant (*p* < 0.05). The unadjusted OR of possible sarcopenia for the 7-year mortality was 3.17 (2.61–3.85) with a statistical difference (*p* < 0.05), but the adjusted OR was 1.11–1.23 without a statistical difference (*p* > 0.05) (**Table 3**).

**Table 3:** Association of sarcopenia with 7-year mortality in the CHARLS.

7-year mortality	n.total	Death event_%	Model 1	*p*-Value	Model 2	*p*-Value	Model 3	*p*-Value	Model 4	*p*-Value
No sarcopenia	3,892	147 (3.8)	1 (Ref)		1 (Ref)		1 (Ref)		1 (Ref)	
Possible sarcopenia	3,570	395 (11.1)	3.17 (2.61–3.85)	<0.001	1.23 (0.98–1.53)	0.073	1.23 (0.98–1.53)	0.075	1.11 (0.89–1.4)	0.356
Sarcopenia	1,125	196 (17.4)	5.37 (4.29–6.74)	<0.001	1.41 (1.07–1.85)	0.015	1.39 (1.06–1.83)	0.019	1.41 (1.06–1.87)	0.017
Severe Sarcopenia	419	133 (31.7)	11.85 (9.1–15.42)	<0.001	2.2 (1.59–3.05)	<0.001	2.1 (1.51–2.92)	<0.001	2.11 (1.51–2.95)	<0.001
Trend. test	9,006	871 (9.7)	2.19 (2.03–2.37)	<0.001	1.27 (1.15–1.4)	<0.001	1.25 (1.13–1.39)	<0.001	1.28 (1.15–1.42)	<0.001

Model 1: crude model. Model 2: adjusted OR for age and sex. Model 3: adjusted OR for age, sex, education level, marriage status, urban area, drinking, and smoking. Model 4: adjusted OR for age, sex, education level, marriage status, urban area, drinking, smoking, diabetes, hypertension, cancer, heart disease, stroke, lung disease, Arthre disease, liver disease, kidney disease, digestive disease, asthma, and memory-related disease.

CHARLS, China Health and Retirement Longitudinal Study; OR, odds ratio.

Over a 7-year follow-up period, stratified analysis by age revealed that among individuals aged <45 years, there were 218 cases (4 deaths) of no sarcopenia, 43 cases (0 deaths) of possible sarcopenia, 3 cases (0 deaths) of sarcopenia, and 1 case (0 deaths) of severe sarcopenia. Among those aged ≥45 and <60 years, there were 3,405 cases (112 deaths) of no sarcopenia, 1,182 cases (52 deaths) of possible sarcopenia, 181 cases (14 deaths) of sarcopenia, and 28 cases (3 deaths) of severe sarcopenia. Among those aged ≥60 and <80 years, there were 263 cases (29 deaths) of no sarcopenia, 2,284 cases (317 deaths) of possible sarcopenia, 864 cases (145 deaths) of sarcopenia, and 325 cases (83 deaths) of severe sarcopenia. Among those aged ≥80 years, there were 6 cases (2 deaths) of no sarcopenia, 61 cases (26 deaths) of possible sarcopenia, 77 cases (37 deaths) of sarcopenia, and 65 cases (47 deaths) of severe sarcopenia. A statistically significant interaction between age and sarcopenia was observed in individuals aged ≥45 years (*p* < 0.05), but not in those aged <45 years (*p* > 0.05). The OR values of sarcopenia and 7-year mortality were 2.94 (95% CI, 1.6–5.39), 1.72 (95% CI, 1.11–2.68), and 5.03 (95% CI, 0.48–52.65) in the 45–60, 60–80, and ≥80 age groups, respectively. The OR values of severe sarcopenia and 7-year mortality were 6.92 (95% CI, 1.95–24.5), 2.59 (95% CI, 1.61–4.17), and 12.52 (95% CI, 1.18–133.18) in the 45–60, 60–80, and ≥80 age groups, respectively (**Table 4**).

**Table 4:** Subgroup analysis stratified by age of the association between sarcopenia and 7-year mortality.

Subgroup	n.total	Death event (%)	Model 1	*p*-Value	Interaction *p*-Value	Model 2	*p*-Value	Interaction *p*-Value	Model 3	*p*-Value	Interaction *p*-Value
**Age < 45 years**					0.026			0.016			0.001
No sarcopenia	218	4 (1.8)	1 (Ref)			1 (Ref)			1 (Ref)		
Possible sarcopenia	43	0 (0)	–	–		–	–		–	–	
Sarcopenia	3	0 (0)	–	–		–	–		–	–	
Severe sarcopenia	1	0 (0)	–	–		–	–		–	–	
Trend test	265	4 (1.5)	–	–		–	–		–	–	
45 years ≥ age < 60 years											
No sarcopenia	3,405	112 (3.3)	1 (Ref)			1 (Ref)			1 (Ref)		
Possible sarcopenia	1,182	52 (4.4)	1.35 (0.97–1.89)	0.078		1.41 (1–1.98)	0.051		1.23 (0.87–1.76)	0.245	
Sarcopenia	181	14 (7.7)	2.46 (1.38–4.39)	0.002		2.69 (1.49–4.85)	0.001		2.94 (1.6–5.39)	<0.001	
Severe sarcopenia	28	3 (10.7)	3.53 (1.05–11.86)	0.042		5.24 (1.52–18.05)	0.009		6.92 (1.95–24.5)	0.003	
Trend test	4,796	181 (3.8)	1.49 (1.2–1.85)	<0.001		1.58 (1.26–1.98)	<0.001		1.56 (1.23–1.98)	<0.001	
60 years ≥ age < 80 years											
No sarcopenia	263	29 (11)	1 (Ref)			1 (Ref)			1 (Ref)		
Possible sarcopenia	2,284	317 (13.9)	1.3 (0.87–1.95)	0.202		1.32 (0.88–1.99)	0.182		1.21 (0.8–1.83)	0.367	
Sarcopenia	864	145 (16.8)	1.63 (1.06–2.49)	0.025		1.71 (1.11–2.64)	0.015		1.72 (1.11–2.68)	0.016	
Severe sarcopenia	325	83 (25.5)	2.77 (1.75–4.38)	<0.001		2.64 (1.65–4.22)	<0.001		2.59 (1.61–4.17)	<0.001	
Trend test	3,736	574 (15.4)	1.39 (1.24–1.56)	<0.001		1.37 (1.22–1.55)	<0.001		1.42 (1.25–1.61)	<0.001	
Age ≥ 80 years											
No sarcopenia	6	2 (33.3)	1 (Ref)			1 (Ref)			1 (Ref)		
Possible sarcopenia	61	26 (42.6)	1.49 (0.25–8.74)	0.661		1.31 (0.22–7.89)	0.768		3.49 (0.33–36.86)	0.298	
Sarcopenia	77	37 (48.1)	1.85 (0.32–10.7)	0.492		1.76 (0.3–10.41)	0.534		5.03 (0.48–52.65)	0.177	
Severe sarcopenia	65	47 (72.3)	5.22 (0.88–31.04)	0.069		4.78 (0.79–28.8)	0.088		12.52 (1.18–133.18)	0.036	
Trend test	209	112 (53.6)	1.81 (1.29–2.54)	0.001		1.83 (1.3–2.59)	0.001		1.97 (1.33–2.91)	0.001	

Model 1: crude model. Model 2: adjusted OR for sex, education level, marriage status, urban area, drinking, and smoking. Model 3: adjusted OR for sex, education level, marriage status, urban area, drinking, smoking, diabetes, hypertension, cancer, heart disease, stroke, lung disease, Arthre disease, liver disease, kidney disease, digestive disease, asthma, and memory-related disease.

OR, odds ratio.

#### The ORs of sarcopenia and severe sarcopenia for 7-year mortality with PSM and IPTW analyses

**Figure 3:**
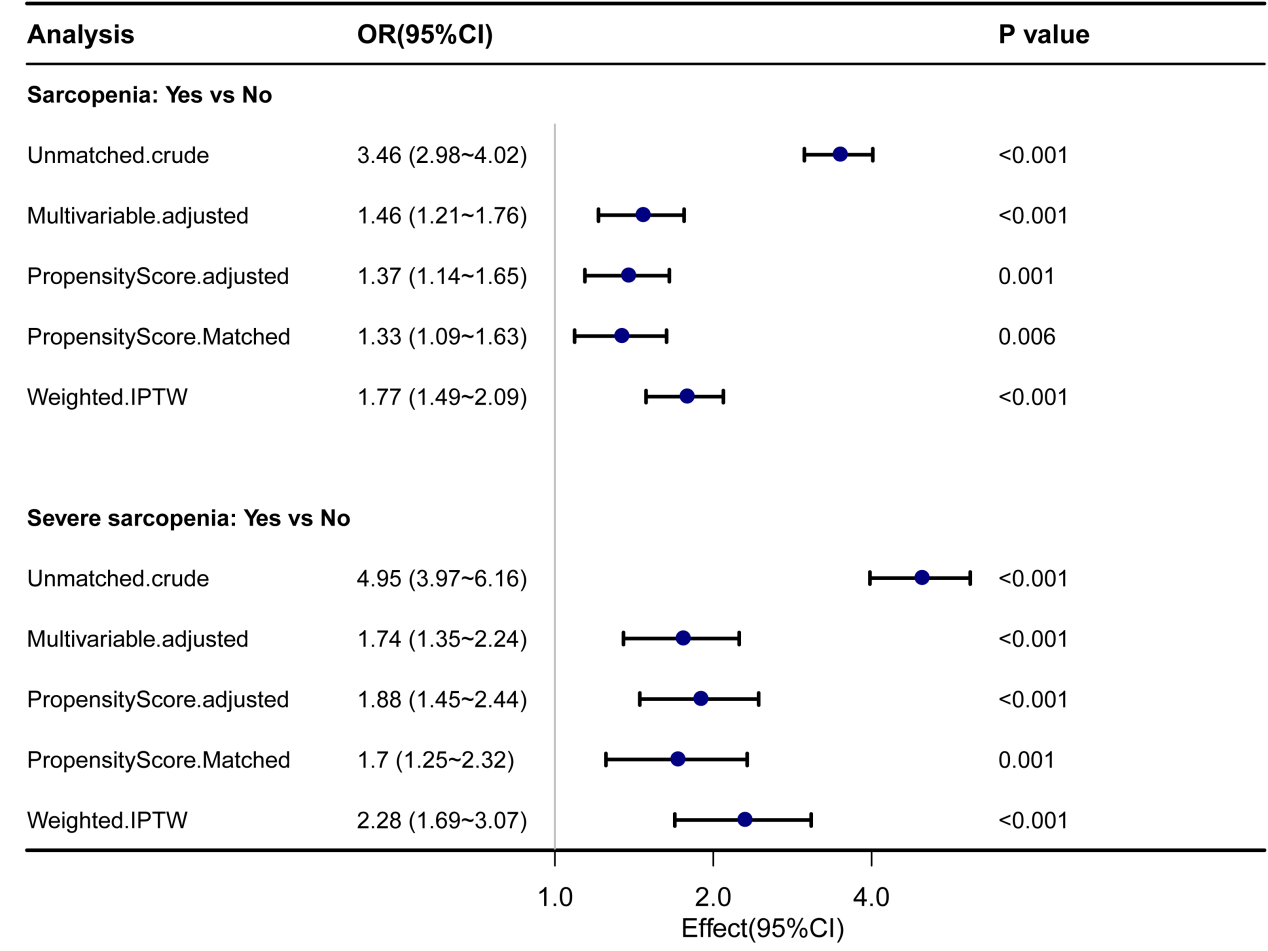
Forest plot shows ORs of all-cause mortality in participants with sarcopenia and severe sarcopenia using propensity score matching analysis. IPTW, the inverse probability of treatment weighting regression analysis; ORs, odds ratios.

The sarcopenia and severe sarcopenia were matched as separate groups for propensity score matching. The baseline characteristics before and after propensity score matching are shown in **Supplementary Table 2**. In both sarcopenia and severe sarcopenia, there were significantly higher OR for 7-year mortality by PSM and IPTW analyses (*p* < 0.05). Before PSM, the unmatched crude ORs for 7-year mortality were 3.46 (2.98–4.02) for sarcopenia and 4.95 (3.97–6.16) for severe sarcopenia, whereas the multivariable ORs were 1.46 (1.21–1.76) for sarcopenia and 1.74 (1.35–2.24) for severe sarcopenia. After propensity score matching, the ORs for 7-year mortality were 1.33 (1.09–1.63) for sarcopenia and 1.7 (1.25–2.32) for severe sarcopenia. After the inverse probability of treatment weighting regression analysis, the ORs for 7-year mortality were 1.77 (1.49–2.09) and 2.28 (1.69–3.07) for sarcopenia and severe sarcopenia, respectively (**Figure 3**).

### Mendelian randomization study

#### The effect of appendicular lean mass on any death

This study examined the role of appendicular lean mass, a key component of sarcopenia, in relation to any death. Analyzing 283 SNPs with MR methods, we found significant results with the MR-Egger method (OR = 1.261, 95% CI, 1.056–1.507, *p* = 0.011). The weighted median method resulted in an OR of 0.948 (95% CI, 0.850–1.058, *p* = 0.341), while the IVW method resulted in an OR of 0.954 (95% CI, 0.892–1.021, *p* = 0.176). Heterogeneity (*p* > 0.05) and potential pleiotropy (*p* < 0.05) were assessed (**Supplementary Figures 1**–**4** and **Table 5**).

**Table 5:** The causal association between sarcopenia and risk of any death.

Exposure	Outcome	nSNP		OR	95% CI	*p*	Heterogeneity test		Pleiotropy_test	
							Cochran’s Q	*p*	Intercept	*p*
Appendicular lean mass	Any death	283	MR-Egger	1.261	1.056–1.507	0.011	245.388	0.980	−0.008	0.001
		283	Weighted median	0.948	0.850–1.058	0.341				
		283	IVW	0.954	0.892–1.021	0.176				
Low hand grip strength (60 years and older) (EWGSOP)	Any death	11	MR-Egger	1.077	0.523–2.218	0.846	14.605	0.147	0.012	0.590
		11	Weighted median	1.168	0.902–1.512	0.238				
		11	IVW	1.310	1.058–1.621	0.013				
Usual walking pace	Any death	52	MR-Egger	0.404	0.057–2.874	0.369	38.768	0.896	0.003	0.697
		52	Weighted median	0.587	0.294–1.173	0.131				
		52	IVW	0.590	0.367–0.950	0.030				

SNP, single-nucleotide polymorphism; MR, Mendelian randomization; IVW, inverse variance weighted; EWGSOP, European Working Group on Sarcopenia in Older People.

#### The effect of low hand grip strength (60 years and older) on any death

In our analysis of the impact of low hand grip strength on any death in individuals aged 60 and older (defined by the European Working Group on Sarcopenia in Older People (EWGSOP) criteria), we employed MR with a dataset of 11 SNPs. The IVW method demonstrated a significant association (OR = 1.310, 95% CI, 1.058–1.621, *p* = 0.013) between low hand grip strength and any death. Subsequently, the MR-Egger method showed an OR of 1.077 (95% CI, 0.523–2.218, *p* = 0.846), and the weighted median method yielded an OR of 1.168 (95% CI, 0.902–1.512, *p* = 0.238). Importantly, there was minimal evidence of heterogeneity (*p* > 0.05), and no substantial pleiotropy was observed (*p* > 0.05) (**Supplementary Figures 5**–**8** and **Table 5**).

#### The effect of the usual walking pace on any death

Examining the influence of the usual walking pace, another integral component of sarcopenia, on any death, 52 SNPs were analyzed using MR methods. The IVW method indicated a significant association (OR = 0.590, 95% CI, 0.367–0.950, *p* = 0.030) between the usual walking pace and any death. In contrast, the MR-Egger method did not reveal a statistically significant effect on mortality (OR = 0.404, 95% CI, 0.057–2.874, *p* = 0.369), and the weighted median method yielded an OR of 0.587 (95% CI, 0.294–1.173, *p* = 0.131). Similar to the previous exposure, minimal evidence of heterogeneity (*p* > 0.05) was observed, and no substantial pleiotropy was detected (*p* > 0.05) (**Supplementary Figures 9**–**12** and **Table 5**).

In summary, these findings reveal associations between sarcopenia components (appendicular lean mass, low hand grip strength, and usual walking pace) and mortality risk.

## Discussion

Our study employed a multifaceted approach by combining longitudinal data from the CHARLS cohort with MR analysis to explore the complex relationships between various components of sarcopenia (appendicular lean mass, low hand grip strength, and usual walking pace) and mortality risk in older adults.

In this study, the prevalence of sarcopenia and severe sarcopenia in our study was 12.5% and 4.7%, respectively, which is consistent with previous studies in China. Our results showed that sarcopenia and severe sarcopenia were associated with a higher risk of 7-year mortality, even after adjusting for several potential confounding factors, consistent with previous studies conducted in other populations (7, 9, 23). Compared to participants without sarcopenia, those with sarcopenia had a 41% higher risk of mortality. Moreover, the risk of mortality was even higher for participants with severe sarcopenia, with a 111% higher risk than those without sarcopenia. This highlights the importance of early detection and intervention for sarcopenia before it progresses to a more severe stage.

Our study also identified several risk factors for mortality, including age, chronic diseases (such as diabetes, hypertension, cancer, stroke, lung disease, and memory-related diseases), and male gender. The association between sarcopenia and mortality risk is more pronounced in older age groups, with the highest risk observed in individuals aged ≥80 years. These findings are consistent with previous studies that have reported an association between these factors and mortality risk (8, 12, 24, 25).

To account for potential confounding by chronic diseases and age, we used propensity score matching, which confirmed the significant association between sarcopenia and mortality risk. While previous research has also utilized this approach, it has mainly focused on examining the relationship between sarcopenia and mortality in the context of a single disease. For instance, Lin et al. demonstrated that patients with both type 2 diabetes and sarcopenia were at higher risk for mortality than those without sarcopenia (13). Similarly, Bang et al. found that sarcopenia was associated with an increased incidence of postoperative acute kidney injury and overall mortality in patients undergoing surgery for abdominal aortic aneurysms (14). Furthermore, several studies have investigated the impact of sarcopenia on postoperative outcomes in cancer patients (11, 26). After utilizing propensity score matching to account for potential confounding factors, the association between sarcopenia and mortality risk remained significant. This underscores the importance of screening for sarcopenia in older adults and implementing age-specific interventions to address associated health risks.

Furthermore, our MR analysis introduced a causal dimension to this association. While MR results did not uniformly establish causal relationships between all sarcopenia components and mortality, they offered valuable insights. Importantly, the MR analysis using the IVW method demonstrated a significant causal association between low hand grip strength in individuals aged 60 and older and mortality risk. Additionally, the MR analysis using the IVW method of walking pace provided further support for this causal relationship. This highlights the importance of muscle strength as a key factor influencing healthy aging and longevity. However, the MR-Egger and weighted median methods did not provide supportive evidence, indicating potential complexities in this relationship and emphasizing the need for further investigation. Meanwhile, the MR analysis did not establish a significant causal relationship between appendicular lean mass and mortality risk. This discrepancy may warrant further exploration, considering the complexity of measuring muscle mass and the multifaceted nature of sarcopenia. It is possible that muscle quality and function, rather than muscle mass alone, play a more critical role in influencing mortality risk. These subtle differences underscore the need for ongoing research to elucidate underlying mechanisms.

Sarcopenia’s impact on mortality extends beyond physical frailty, encompassing a range of physiological and metabolic changes (8). One critical aspect is the increased risk of falls and fractures among individuals with reduced muscle mass and strength (27, 28). These incidents can trigger a chain reaction, leading to complications like infections, immobility, and secondary muscle loss, ultimately contributing to mortality (7, 8, 28). Chronic inflammation, hormonal shifts, and malnutrition are pivotal factors linking sarcopenia to mortality (25, 29). They can initiate or worsen various chronic diseases, significantly elevating the risk of premature death (10, 13). Strategies aimed at managing chronic diseases, reducing inflammation, optimizing hormone levels, and ensuring adequate nutrition can collectively improve the overall wellbeing of sarcopenic individuals, potentially reducing their mortality risk.

Our study has several strengths, including a large sample size, a longitudinal design, and the use of standardized diagnostic criteria for sarcopenia. Additionally, the inclusion of Mendelian randomization analysis, employing multiple methods such as IVW, MR-Egger, and weighted median, provided a robust basis for establishing causal links between sarcopenia components and mortality. Finally, rigorous adjustments for sociodemographic and medical covariates were performed to mitigate potential confounding effects. However, our study also has some limitations. First, the diagnosis of sarcopenia was based on a single measurement of muscle mass, strength, and function, which may not accurately reflect the individual’s sarcopenia status over time. Second, we did not have data on the cause of death, which limits our ability to assess the specific causes of mortality associated with sarcopenia. Finally, our study was based on observational data from Chinese adults, and the generalizability of our findings to other populations may be limited. Additionally, while efforts were made to address pleiotropy, the presence of residual pleiotropic effects cannot be entirely excluded.

In conclusion, our study demonstrates that sarcopenia and severe sarcopenia are strongly linked to higher mortality risk. Further research should explore interventions to mitigate these risks and enhance outcomes for individuals with sarcopenia.

## Data availability statement

Publicly available datasets were analyzed in this study. After obtaining permission, the CHARLS data can be found at: https://charls.charlsdata.com/users/profile/index/zh-cn.html.

## Ethics statement 

The CHARLS is approved by the Biomedical Ethics Review Committee of Peking University, and all participants provide informed consent. The data of the CHARLS are available for free on the Peking University Open Research Data Platform (https://charls.charlsdata.com/). The study used publicly available deidentified data, and informed consent was waived. Based on a publicly accessible database, this study did not require ethical approval or informed consent.

## Author contributions

LX: conception of the protocol, data analysis and interpretation, acquisition of the data, statistical analysis and interpretation of the data, and manuscript preparation. TL: manuscript preparation. TG, ZZ, SW, XHW, XYW, JZ, PZ, YL, and LL: study concept and design and interpretation of the results. GY, YL, LL, JZ, and PZ: revision of the manuscript. SY, LK, and ZL: concept and design, final drafting of the manuscript, and study supervision. All authors agreed to be fully accountable for ensuring the integrity and accuracy of the work. All authors contributed to the article and approved the submitted version.

## Supplementary material

Supplementary Figure 1 The scatter plot of Appendicular Lean Mass on any death.

Supplementary Figure 2 The forest plot of Appendicular Lean Mass on any death.

Supplementary Figure 3 The leaveoneout plot of Appendicular Lean Mass on any death.

Supplementary Figure 4 The funnel plot of Appendicular Lean Mass on any death.

Supplementary Figure 5 The scatter plot of Low Hand Grip Strength (60 years and older) on any death.

Supplementary Figure 6 The forest plot of Low Hand Grip Strength (60 years and older) on any death.

Supplementary Figure 7 The leaveoneout plot of Low Hand Grip Strength (60 years and older) on any death.

Supplementary Figure 8 The funnel plot of Low Hand Grip Strength (60 years and older) on any death.

Supplementary Figure 9 The scatter plot of the Usual Walking Pace on any death.

Supplementary Figure 10 The forest plot of the Usual Walking Pace on any death.

Supplementary Figure 11 The leaveoneout plot of the Usual Walking Pace on any death.

Supplementary Figure 12 The funnel plot of the Usual Walking Pace on any death.
