# Supplementary figures and images for: The relationship between sarcopenia and mortality in Chinese community-dwelling adults: a 7-year cohort study with propensity score matching and Mendelian randomization
Source: Front Endocrinol (Lausanne). 2023 Oct 4;14:1215512. doi: 10.3389/fendo.2023.1215512 (PMC10582747; doi:10.3389/fendo.2023.1215512)

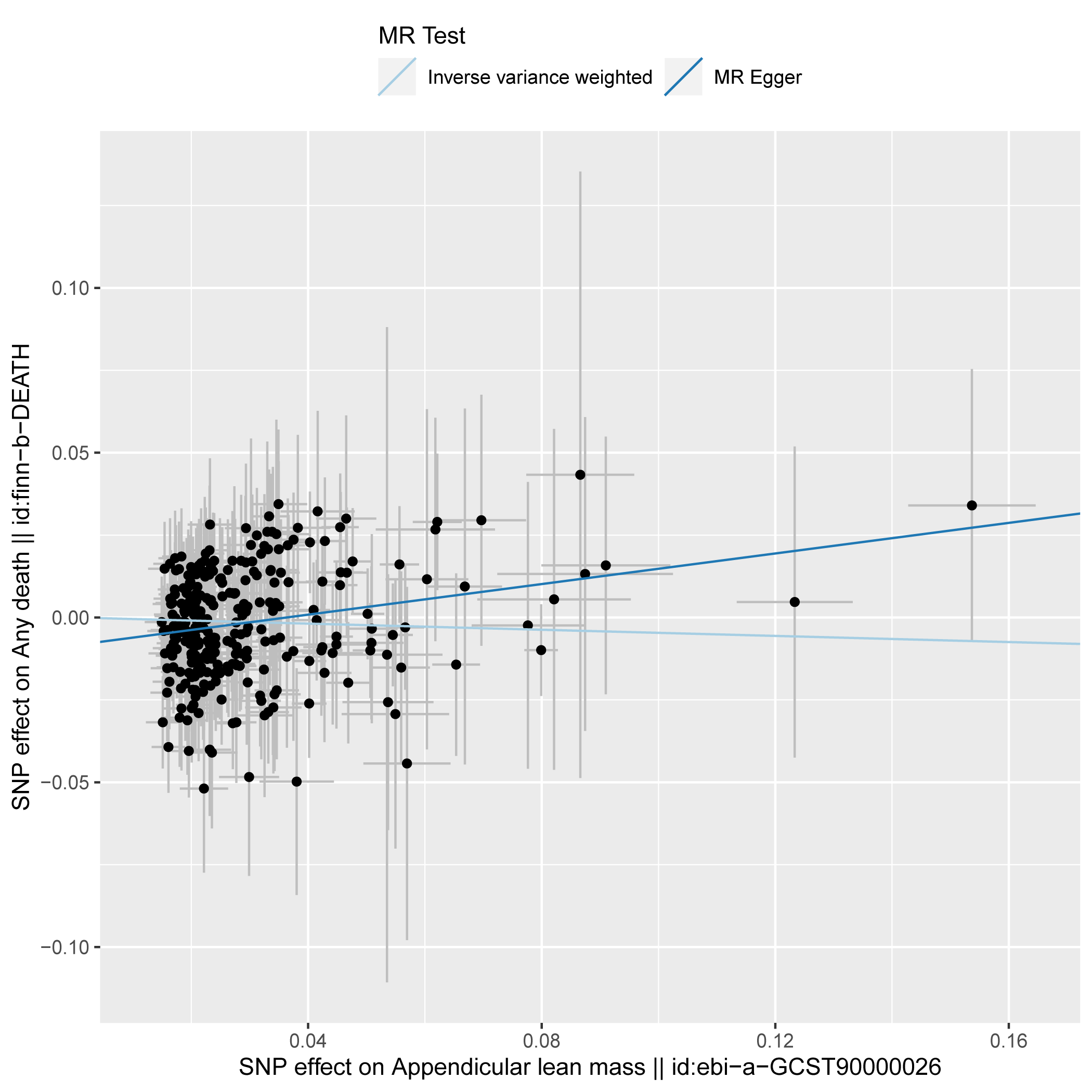

Supplement: Supplementary Figure 1 — The scatter plot of Appendicular Lean Mass on any death. [file Image_1.tif]

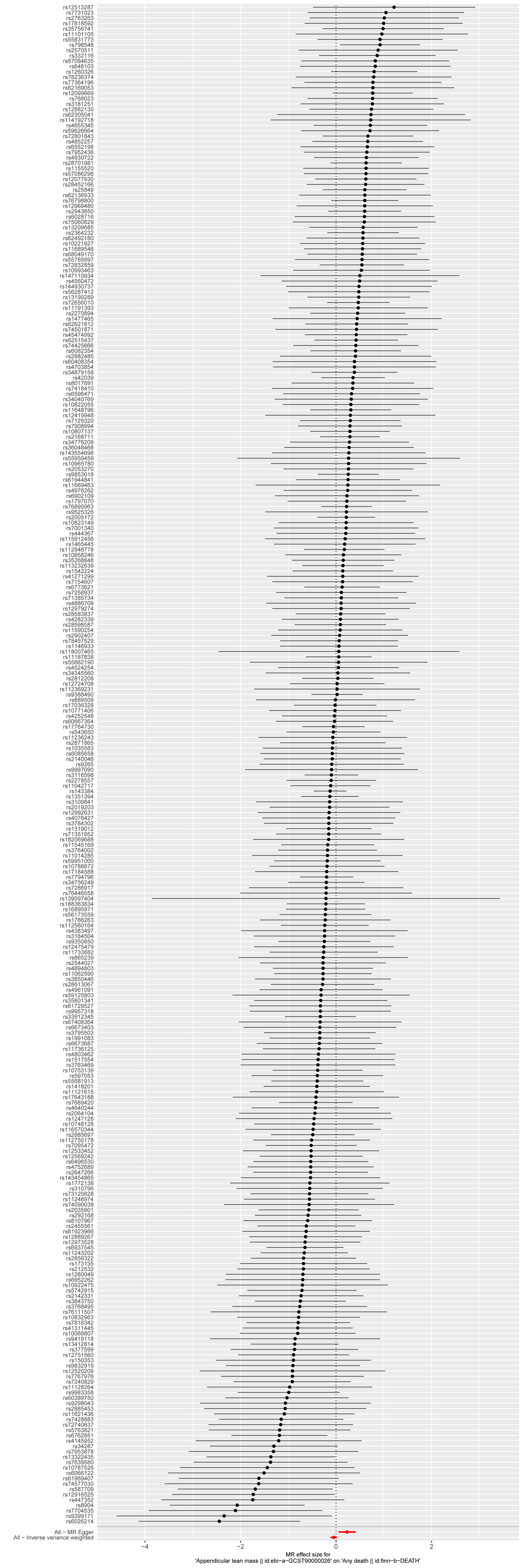

Supplement: Supplementary Figure 2 — The forest plot of Appendicular Lean Mass on any death. [file Image_2.tif]

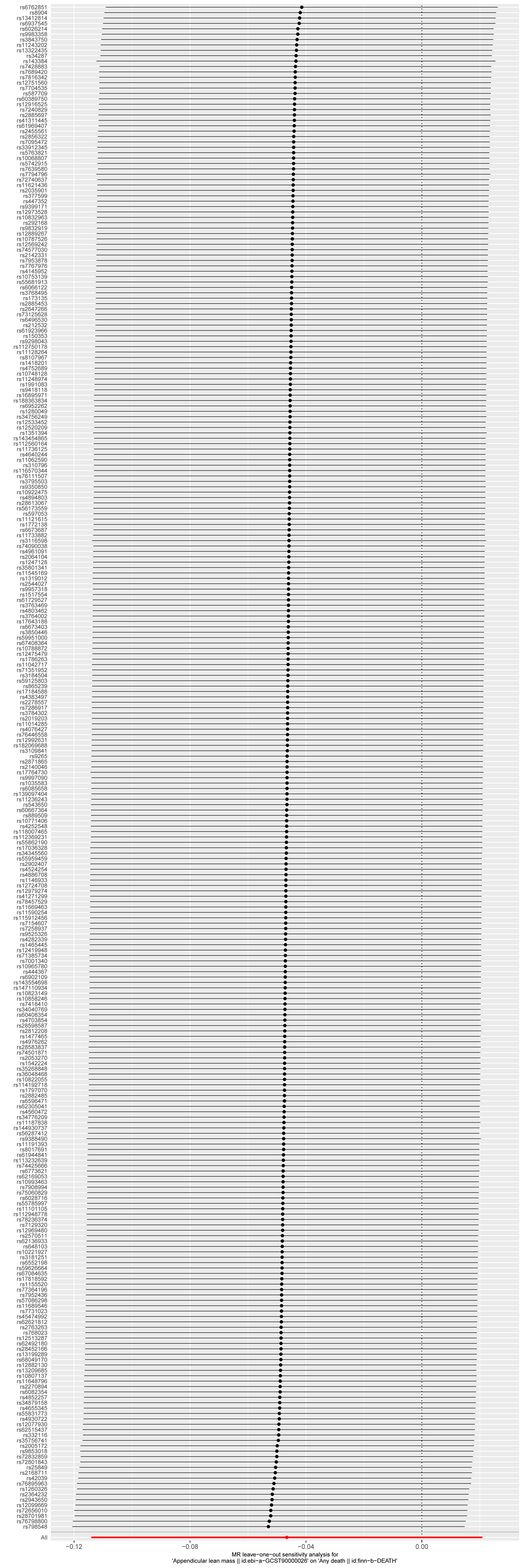

Supplement: Supplementary Figure 3 — The leaveoneout plot of Appendicular Lean Mass on any death. [file Image_3.tif]

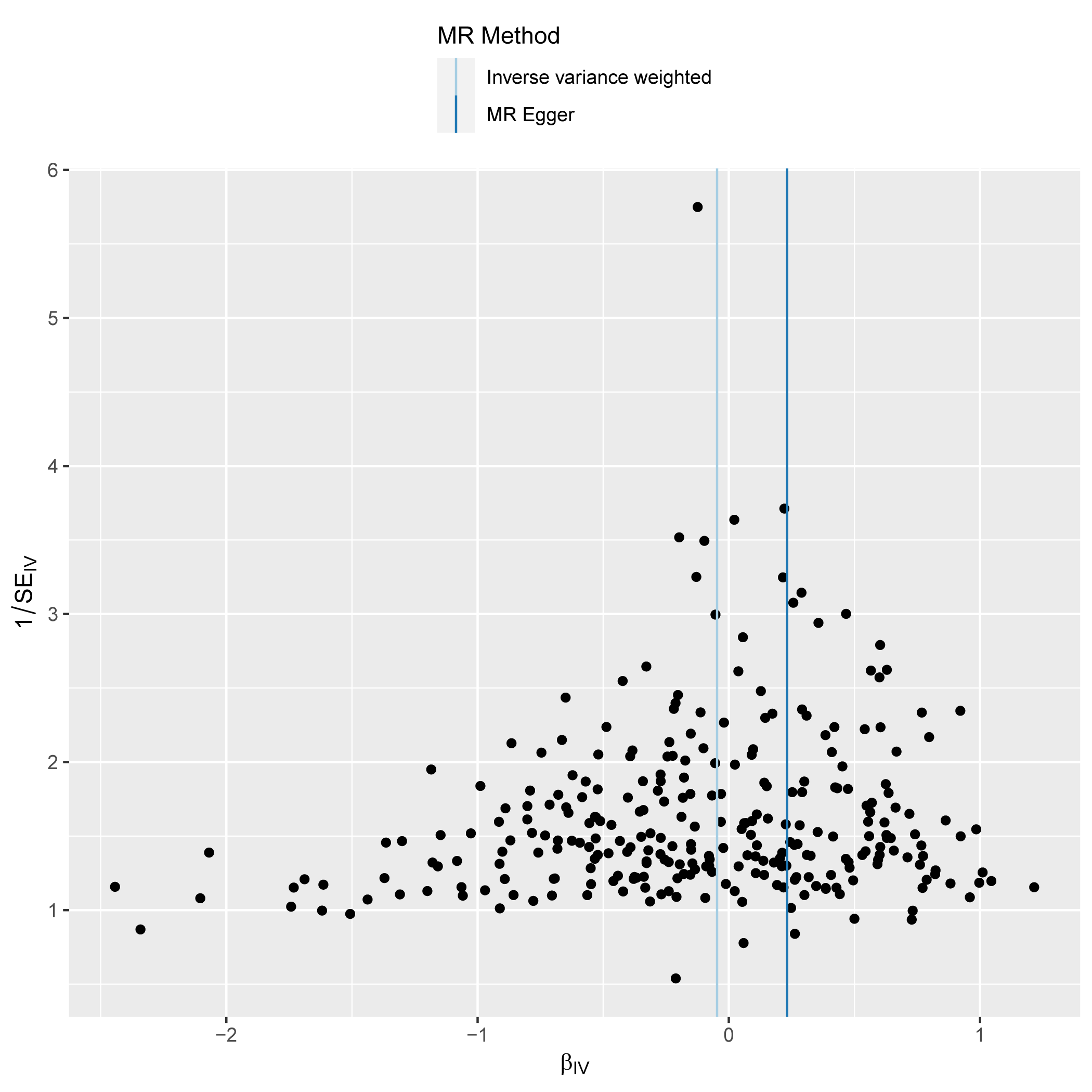

Supplement: Supplementary Figure 4 — The funnel plot of Appendicular Lean Mass on any death. [file Image_4.tif]

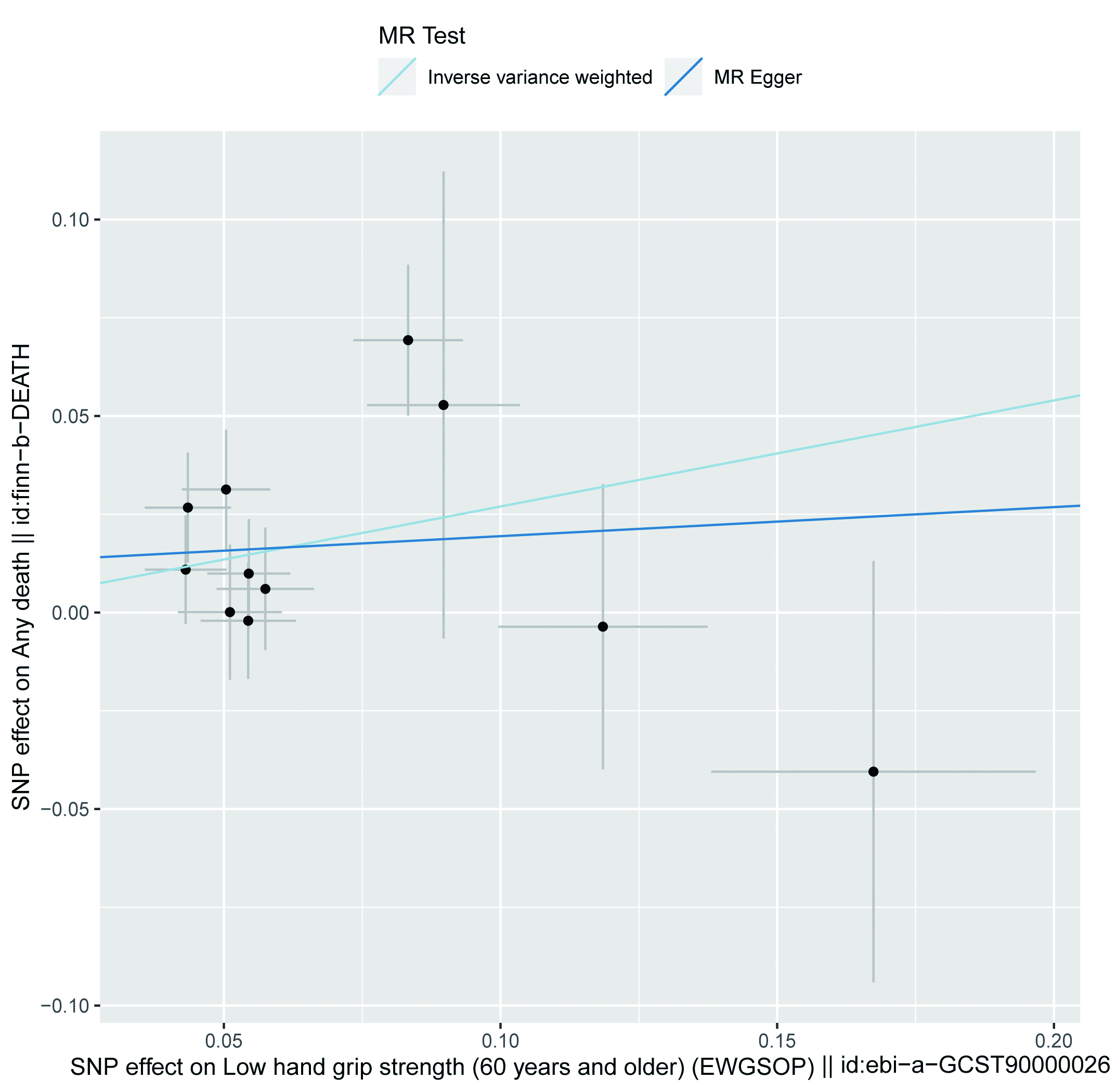

Supplement: Supplementary Figure 5 — The scatter plot of Low Hand Grip Strength (60 years and older) on any death. [file Image_5.tif]

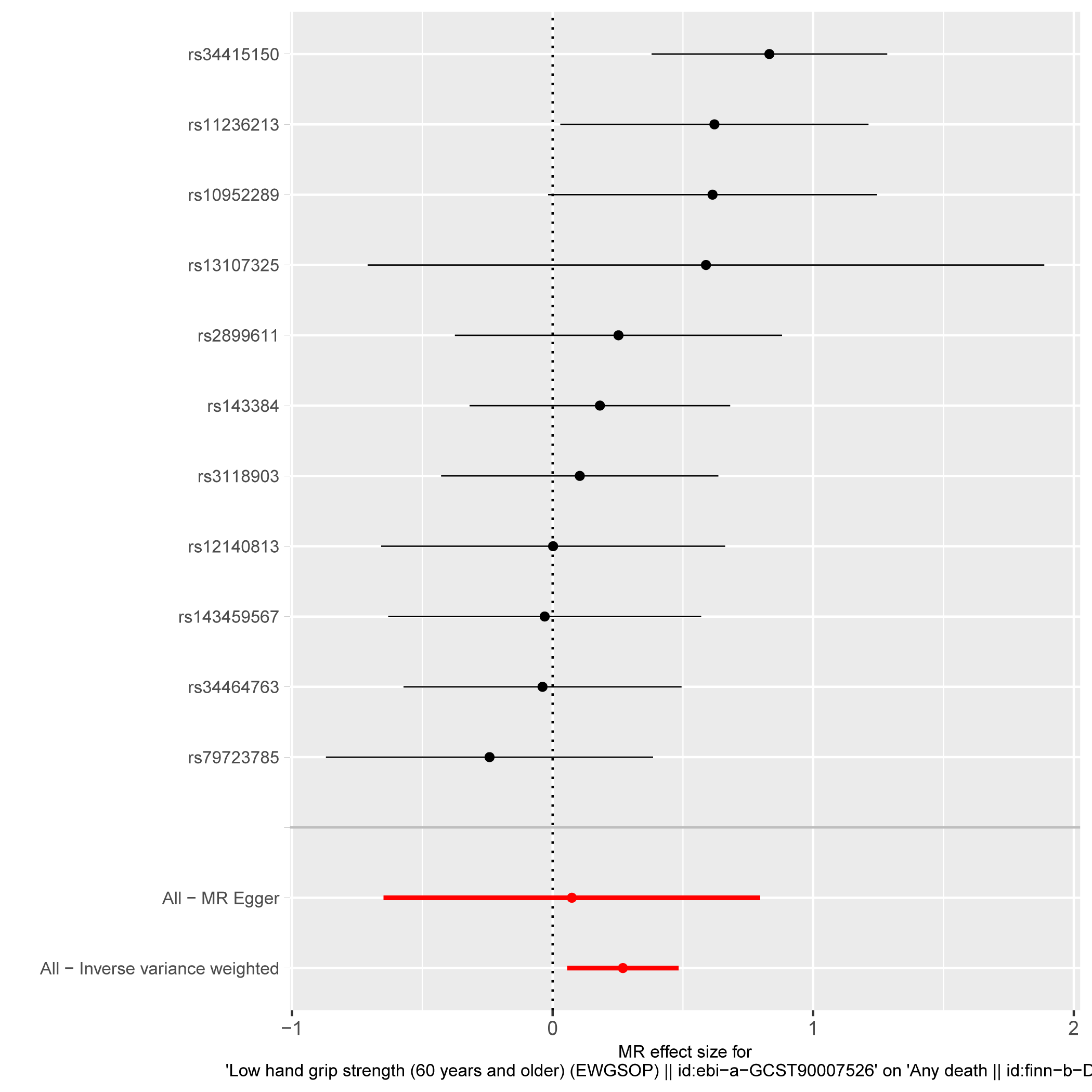

Supplement: Supplementary Figure 6 — The forest plot of Low Hand Grip Strength (60 years and older) on any death. [file Image_6.tif]

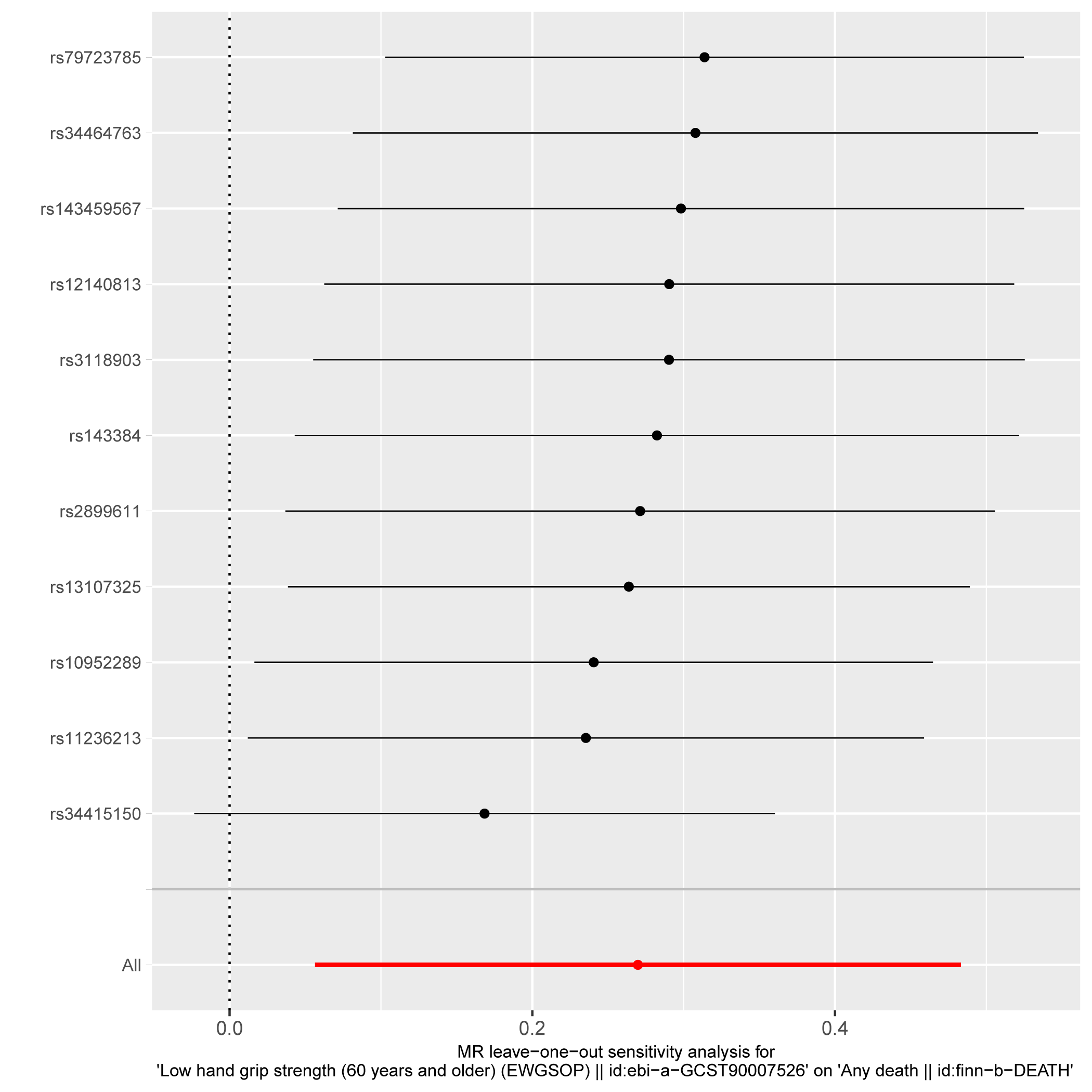

Supplement: Supplementary Figure 7 — The leaveoneout plot of Low Hand Grip Strength (60 years and older) on any death. [file Image_7.tif]

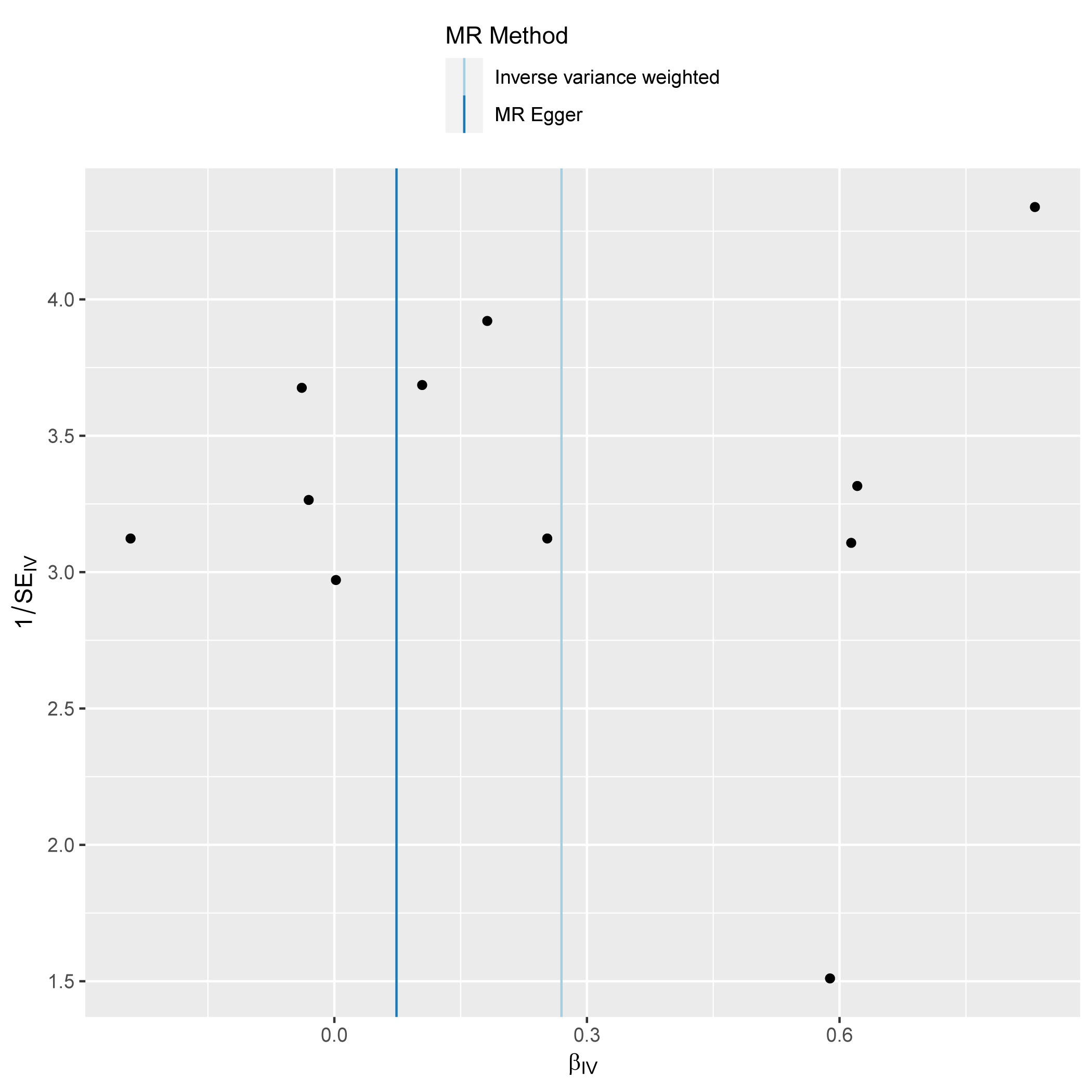

Supplement: Supplementary Figure 8 — The funnel plot of Low Hand Grip Strength (60 years and older) on any death. [file Image_8.tif]

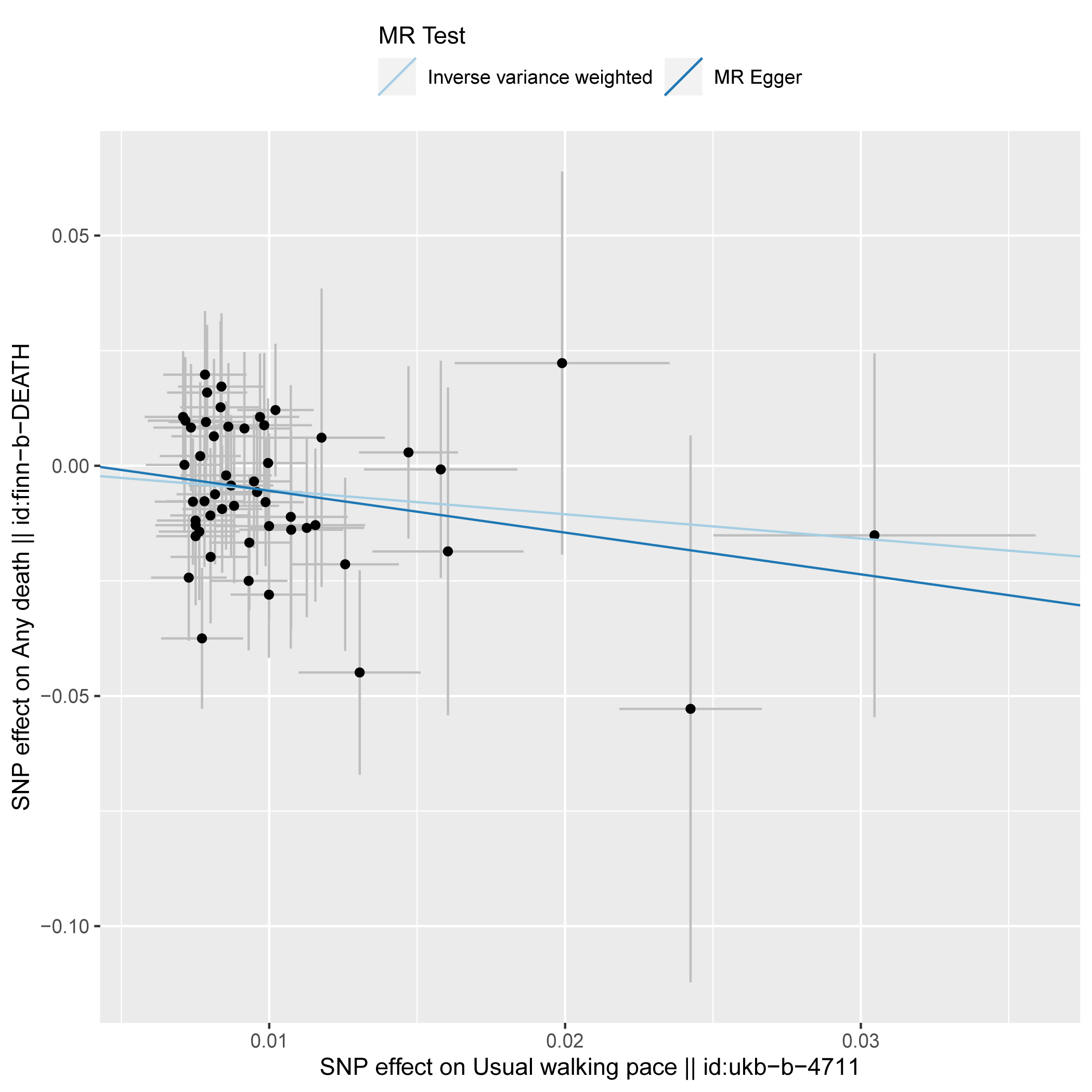

Supplement: Supplementary Figure 9 — The scatter plot of the Usual Walking Pace on any death. [file Image_9.tif]

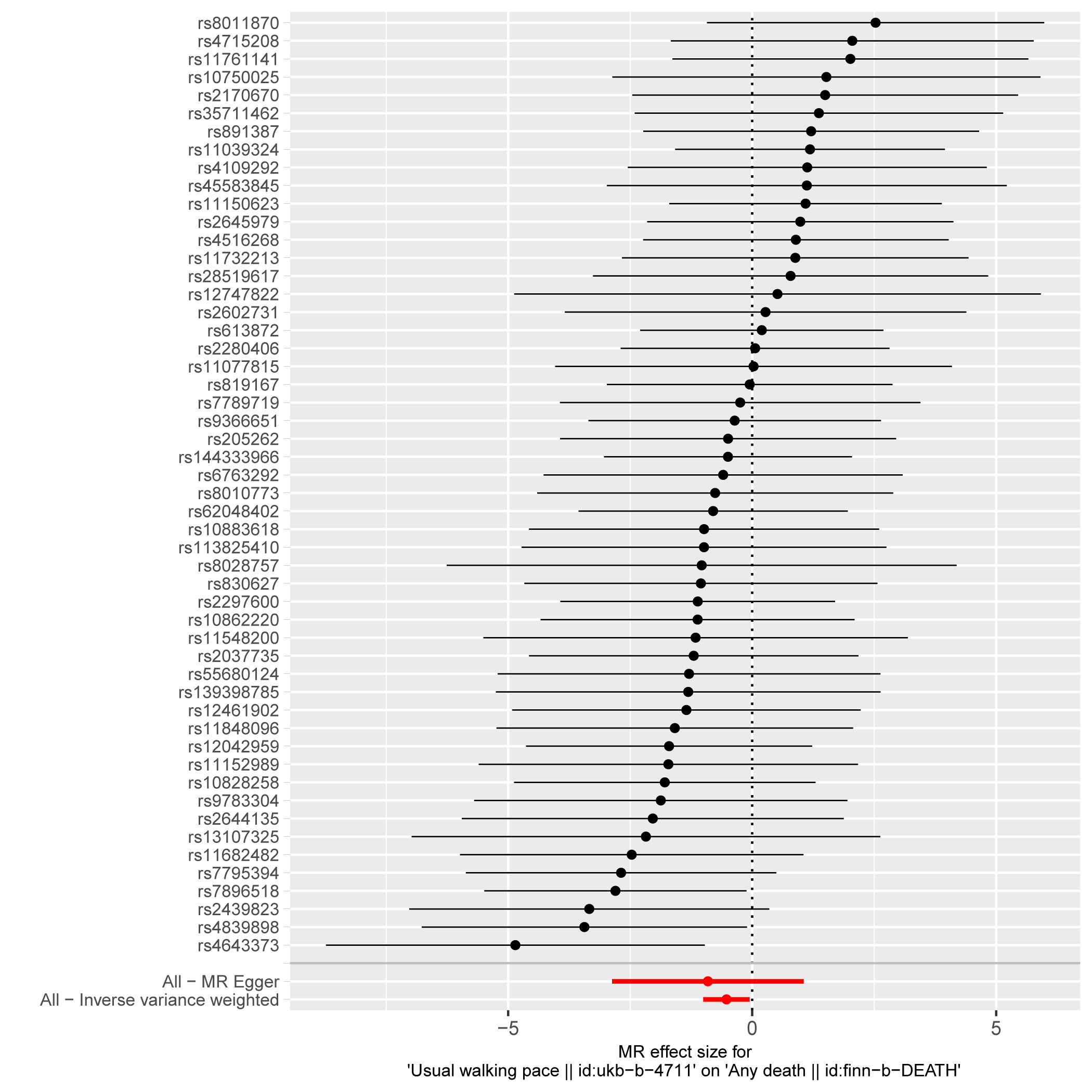

Supplement: Supplementary Figure 10 — The forest plot of the Usual Walking Pace on any death. [file Image_10.tif]

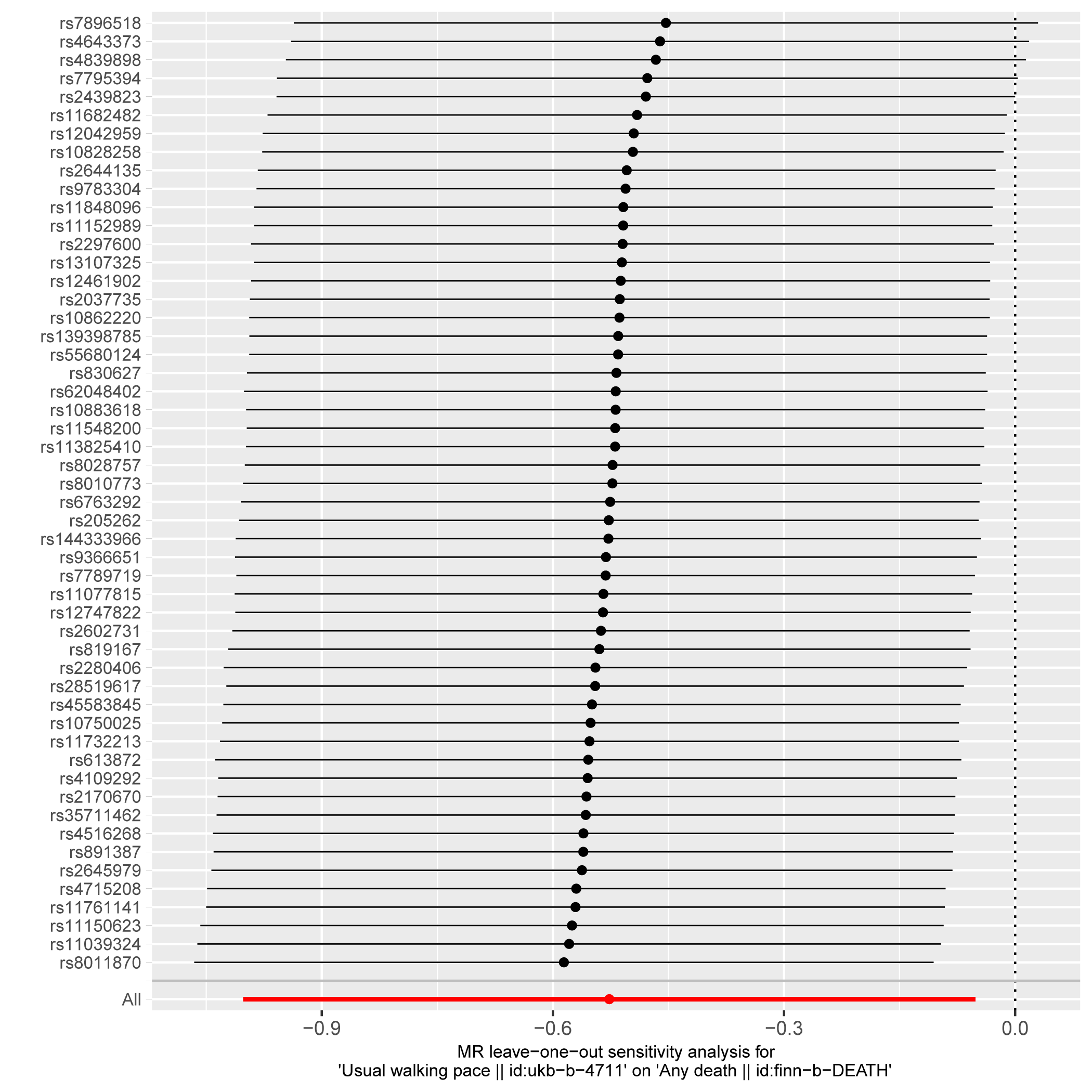

Supplement: Supplementary Figure 11 — The leaveoneout plot of the Usual Walking Pace on any death. [file Image_11.tif]

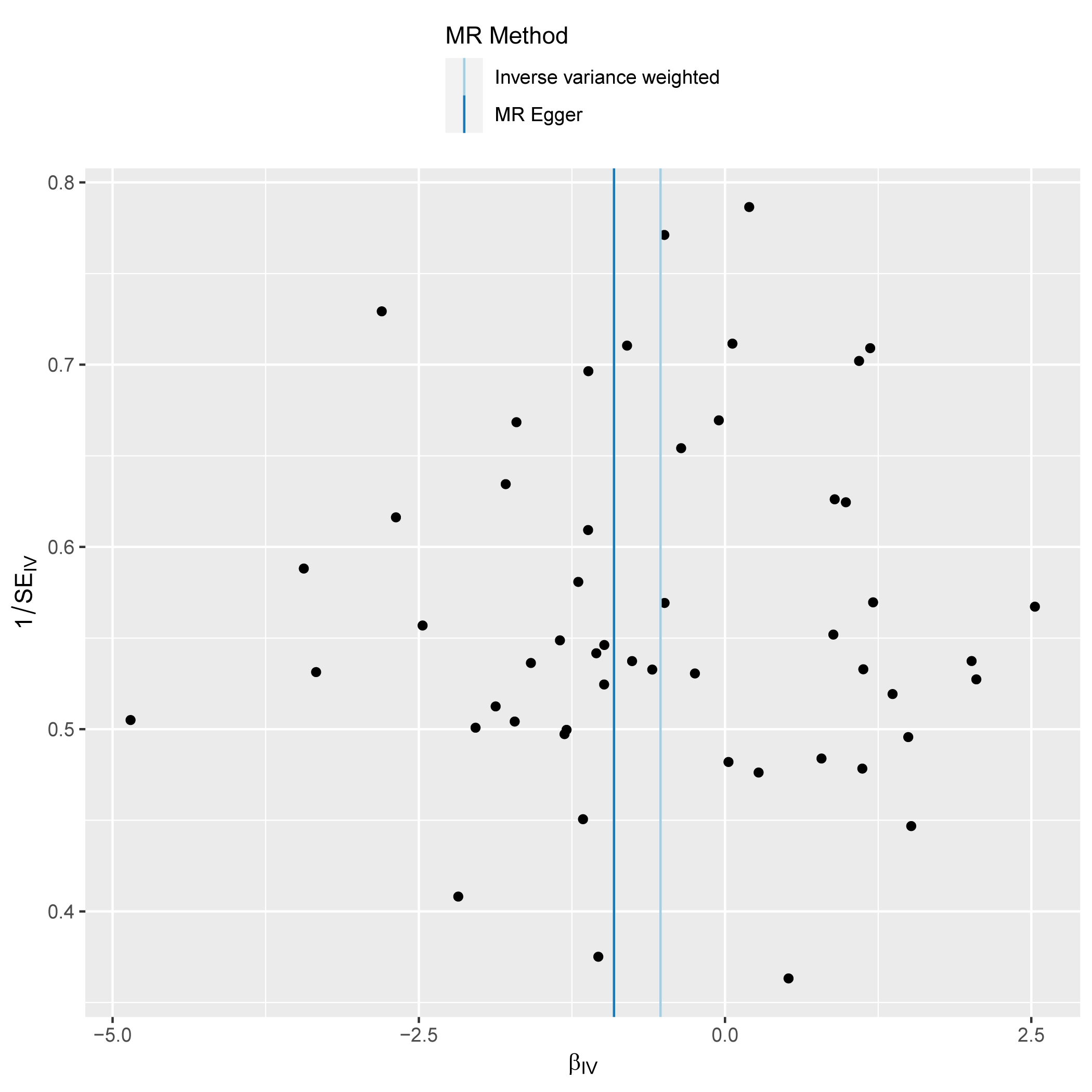

Supplement: Supplementary Figure 12 — The funnel plot of the Usual Walking Pace on any death. [file Image_12.tif]
