# Supplementary material for: The relationship between sarcopenia and mortality in Chinese community-dwelling adults: a 7-year cohort study with propensity score matching and Mendelian randomization
Source: Front Endocrinol (Lausanne). 2023 Oct 4;14:1215512. doi: 10.3389/fendo.2023.1215512 (PMC10582747; doi:10.3389/fendo.2023.1215512)
Supplement: Supplementary file 13 [file Table_1.docx]

**Supplementary Table 1**. Detailed description of the GWAS data involved in this study.

| Phenotype | Races | Sample size | Years | No. of SNPs | Website |
| --- | --- | --- | --- | --- | --- |
| Appendicular lean mass | European | 205,513 | 2020 | 18,164,071 | <https://gwas.mrcieu.ac.uk/datasets/ebi-a-GCST90000026/> |
| Low hand grip strength (60 years and older) (EWGSOP) | European | 256,523 | 2021 | 9,336,415 | https://gwas.mrcieu.ac.uk/datasets/ebi-a-GCST90007526/ |
| Usual walking pace | European | 459,915 | 2018 | 9,851,867 | <https://gwas.mrcieu.ac.uk/datasets/ukb-b-4711/> |
| Any death | European | 218,792 | 2021 | 16,380,466 | <https://gwas.mrcieu.ac.uk/datasets/finn-b-DEATH/> |

**Supplementary Table 2.** Characteristics of participants with sarcopenia and severe sarcopenia before and after Propensity-Score–Matched

| Characteristics | Before matched Sarcopenia | | |  | Matched Sarcopenia | | |  | Before matched Severe Sarcopenia | | |  | Matched Severe Sarcopenia | | |
| --- | --- | --- | --- | --- | --- | --- | --- | --- | --- | --- | --- | --- | --- | --- | --- |
|  | No Sarcopenia  (n = 7462) | Sarcopenia  (n = 1544) | *P value* |  | No Sarcopenia  (n = 1365) | Sarcopenia  (n = 1365) | P value |  | No Severe Sarcopenia  (n = 7462) | Severe Sarcopenia  (n = 1544) | P value |  | No Severe Sarcopenia  (n = 416) | Severe Sarcopenia  (n = 416) | P value |
| Age, y | 56.9 ± 8.7 | 68.3 ± 8.4 | < 0.001 |  | 66.8 ± 8.0 | 66.8 ± 7.6 | 0.871 |  | 56.9 ± 8.7 | 68.3 ± 8.4 | < 0.001 |  | 71.2 ± 8.2 | 71.3 ± 8.0 | 0.818 |
| Gender, n (%) |  |  | < 0.001 |  |  |  | 0.487 |  |  |  | < 0.001 |  |  |  | 0.676 |
| Male | 3491 (46.8) | 647 (41.9) |  |  | 603 (44.2) | 585 (42.9) |  |  | 3491 (46.8) | 647 (41.9) |  |  | 190 (45.7) | 184 (44.2) |  |
| Female | 3971 (53.2) | 897 (58.1) |  |  | 762 (55.8) | 780 (57.1) |  |  | 3971 (53.2) | 897 (58.1) |  |  | 226 (54.3) | 232 (55.8) |  |
| Education, n (%) |  |  | < 0.001 |  |  |  | 0.351 |  |  |  | < 0.001 |  |  |  | 0.238 |
| Elementary school or below | 5034 (67.5) | 1378 (89.2) |  |  | 1222 (89.5) | 1199 (87.8) |  |  | 5034 (67.5) | 1378 (89.2) |  |  | 402 (96.6) | 393 (94.5) |  |
| Secondary school | 2346 (31.4) | 160 (10.4) |  |  | 139 (10.2) | 160 (11.7) |  |  | 2346 (31.4) | 160 (10.4) |  |  | 13 (3.1) | 22 (5.3) |  |
| College or above | 82 (1.1) | 6 (0.4) |  |  | 4 (0.3) | 6 (0.4) |  |  | 82 (1.1) | 6 (0.4) |  |  | 1 (0.2) | 1 (0.2) |  |
| Marriage, n (%) |  |  | < 0.001 |  |  |  | 0.705 |  |  |  | < 0.001 |  |  |  | 0.369 |
| Single | 40 (0.5) | 17 (1.1) |  |  | 11 (0.8) | 15 (1.1) |  |  | 40 (0.5) | 17 (1.1) |  |  | 9 (2.2) | 9 (2.2) |  |
| Married | 6746 (90.4) | 1161 (75.2) |  |  | 1077 (78.9) | 1068 (78.2) |  |  | 6746 (90.4) | 1161 (75.2) |  |  | 304 (73.1) | 286 (68.8) |  |
| Divorced or widowed or others | 676 (9.1) | 366 (23.7) |  |  | 277 (20.3) | 282 (20.7) |  |  | 676 (9.1) | 366 (23.7) |  |  | 103 (24.8) | 121 (29.1) |  |
| Area, n (%) |  |  | < 0.001 |  |  |  | 0.621 |  |  |  | < 0.001 |  |  |  | 0.14 |
| Rural area | 2615 (35) | 357 (23.1) |  |  | 320 (23.4) | 331 (24.2) |  |  | 2615 (35) | 357 (23.1) |  |  | 88 (21.2) | 106 (25.5) |  |
| Urban area | 4847 (65) | 1187 (76.9) |  |  | 1045 (76.6) | 1034 (75.8) |  |  | 4847 (65) | 1187 (76.9) |  |  | 328 (78.8) | 310 (74.5) |  |
| Drinking, n (%) |  |  | < 0.001 |  |  |  | 0.495 |  |  |  | < 0.001 |  |  |  | 0.866 |
| No | 5330 (71.4) | 1213 (78.6) |  |  | 1044 (76.5) | 1059 (77.6) |  |  | 5330 (71.4) | 1213 (78.6) |  |  | 325 (78.1) | 327 (78.6) |  |
| Yes | 2132 (28.6) | 331 (21.4) |  |  | 321 (23.5) | 306 (22.4) |  |  | 2132 (28.6) | 331 (21.4) |  |  | 91 (21.9) | 89 (21.4) |  |
| Smoking, n (%) |  |  | 0.53 |  |  |  | 0.741 |  |  |  | 0.53 |  |  |  | 0.552 |
| No | 5236 (70.2) | 1071 (69.4) |  |  | 938 (68.7) | 946 (69.3) |  |  | 5236 (70.2) | 1071 (69.4) |  |  | 279 (67.1) | 287 (69) |  |
| Yes | 2226 (29.8) | 473 (30.6) |  |  | 427 (31.3) | 419 (30.7) |  |  | 2226 (29.8) | 473 (30.6) |  |  | 137 (32.9) | 129 (31) |  |
| Diabetes, n (%) |  |  | < 0.001 |  |  |  | 0.662 |  |  |  | < 0.001 |  |  |  | 0.712 |
| No | 6160 (82.6) | 1335 (86.5) |  |  | 1166 (85.4) | 1174 (86) |  |  | 6160 (82.6) | 1335 (86.5) |  |  | 347 (83.4) | 343 (82.5) |  |
| Yes | 1302 (17.4) | 209 (13.5) |  |  | 199 (14.6) | 191 (14) |  |  | 1302 (17.4) | 209 (13.5) |  |  | 69 (16.6) | 73 (17.5) |  |
| Hypertension, n (%) |  |  | < 0.001 |  |  |  | 0.819 |  |  |  | < 0.001 |  |  |  | 0.28 |
| No | 5410 (72.5) | 1230 (79.7) |  |  | 1056 (77.4) | 1061 (77.7) |  |  | 5410 (72.5) | 1230 (79.7) |  |  | 316 (76) | 329 (79.1) |  |
| Yes | 2052 (27.5) | 314 (20.3) |  |  | 309 (22.6) | 304 (22.3) |  |  | 2052 (27.5) | 314 (20.3) |  |  | 100 (24) | 87 (20.9) |  |
| Cancer, n (%) |  |  | 0.323 |  |  |  | 0.827 |  |  |  | 0.323 |  |  |  | 1 |
| No | 7389 (99) | 1533 (99.3) |  |  | 1354 (99.2) | 1355 (99.3) |  |  | 7389 (99) | 1533 (99.3) |  |  | 414 (99.5) | 413 (99.3) |  |
| Yes | 73 (1) | 11 (0.7) |  |  | 11 (0.8) | 10 (0.7) |  |  | 73 (1) | 11 (0.7) |  |  | 2 (0.5) | 3 (0.7) |  |
| Heart disease, n (%) |  |  | 0.084 |  |  |  | 0.67 |  |  |  | 0.084 |  |  |  | 0.746 |
| No | 6568 (88) | 1383 (89.6) |  |  | 1210 (88.6) | 1217 (89.2) |  |  | 6568 (88) | 1383 (89.6) |  |  | 366 (88) | 369 (88.7) |  |
| Yes | 894 (12) | 161 (10.4) |  |  | 155 (11.4) | 148 (10.8) |  |  | 894 (12) | 161 (10.4) |  |  | 50 (12) | 47 (11.3) |  |
| Stroke, n (%) |  |  | 0.495 |  |  |  | 0.633 |  |  |  | 0.495 |  |  |  | 1 |
| No | 7272 (97.5) | 1500 (97.2) |  |  | 1331 (97.5) | 1327 (97.2) |  |  | 7272 (97.5) | 1500 (97.2) |  |  | 398 (95.7) | 398 (95.7) |  |
| Yes | 190 (2.5) | 44 (2.8) |  |  | 34 (2.5) | 38 (2.8) |  |  | 190 (2.5) | 44 (2.8) |  |  | 18 (4.3) | 18 (4.3) |  |
| Lung disease, n (%) |  |  | < 0.001 |  |  |  | 0.522 |  |  |  | < 0.001 |  |  |  | 0.704 |
| No | 6835 (91.6) | 1296 (83.9) |  |  | 1152 (84.4) | 1164 (85.3) |  |  | 6835 (91.6) | 1296 (83.9) |  |  | 352 (84.6) | 348 (83.7) |  |
| Yes | 627 (8.4) | 248 (16.1) |  |  | 213 (15.6) | 201 (14.7) |  |  | 627 (8.4) | 248 (16.1) |  |  | 64 (15.4) | 68 (16.3) |  |
| Arthre disease, n (%) |  |  | 0.085 |  |  |  | 0.724 |  |  |  | 0.085 |  |  |  | 1 |
| No | 4874 (65.3) | 973 (63) |  |  | 830 (60.8) | 839 (61.5) |  |  | 4874 (65.3) | 973 (63) |  |  | 250 (60.1) | 250 (60.1) |  |
| Yes | 2588 (34.7) | 571 (37) |  |  | 535 (39.2) | 526 (38.5) |  |  | 2588 (34.7) | 571 (37) |  |  | 166 (39.9) | 166 (39.9) |  |
| Liver disease, n (%) |  |  | 0.964 |  |  |  | 0.582 |  |  |  | 0.964 |  |  |  | 0.806 |
| No | 7306 (97.9) | 1512 (97.9) |  |  | 1340 (98.2) | 1336 (97.9) |  |  | 7306 (97.9) | 1512 (97.9) |  |  | 408 (98.1) | 407 (97.8) |  |
| Yes | 156 (2.1) | 32 (2.1) |  |  | 25 (1.8) | 29 (2.1) |  |  | 156 (2.1) | 32 (2.1) |  |  | 8 (1.9) | 9 (2.2) |  |
| Kidney disease, n (%) | |  | 0.498 |  |  |  | 0.734 |  |  |  | 0.498 |  |  |  | 0.646 |
| No | 7033 (94.3) | 1462 (94.7) |  |  | 1294 (94.8) | 1290 (94.5) |  |  | 7033 (94.3) | 1462 (94.7) |  |  | 395 (95) | 392 (94.2) |  |
| Yes | 429 (5.7) | 82 (5.3) |  |  | 71 (5.2) | 75 (5.5) |  |  | 429 (5.7) | 82 (5.3) |  |  | 21 (5) | 24 (5.8) |  |
| Digest disease, n (%) |  |  | 0.011 |  |  |  | 0.314 |  |  |  | 0.011 |  |  |  | 0.488 |
| No | 5801 (77.7) | 1154 (74.7) |  |  | 1003 (73.5) | 1026 (75.2) |  |  | 5801 (77.7) | 1154 (74.7) |  |  | 294 (70.7) | 303 (72.8) |  |
| Yes | 1661 (22.3) | 390 (25.3) |  |  | 362 (26.5) | 339 (24.8) |  |  | 1661 (22.3) | 390 (25.3) |  |  | 122 (29.3) | 113 (27.2) |  |
| Asthma, n (%) |  |  | < 0.001 |  |  |  | 0.497 |  |  |  | < 0.001 |  |  |  | 0.32 |
| No | 7159 (95.9) | 1432 (92.7) |  |  | 1266 (92.7) | 1275 (93.4) |  |  | 7159 (95.9) | 1432 (92.7) |  |  | 393 (94.5) | 386 (92.8) |  |
| Yes | 303 (4.1) | 112 (7.3) |  |  | 99 (7.3) | 90 (6.6) |  |  | 303 (4.1) | 112 (7.3) |  |  | 23 (5.5) | 30 (7.2) |  |
| Memory-related disease, n (%) | |  | 0.016 |  |  |  | 0.791 |  |  |  | 0.016 |  |  |  | 0.583 |
| No | 7363 (98.7) | 1511 (97.9) |  |  | 1337 (97.9) | 1335 (97.8) |  |  | 7363 (98.7) | 1511 (97.9) |  |  | 399 (95.9) | 402 (96.6) |  |
| Yes | 99 (1.3) | 33 (2.1) |  |  | 28 (2.1) | 30 (2.2) |  |  | 99 (1.3) | 33 (2.1) |  |  | 17 (4.1) | 14 (3.4) |  |
